# *Trans*-Chalcone alleviates overt pain-like behavior by targeting the activation of nociceptive neuron TRPV1 and TRPA1 channels

**DOI:** 10.1007/s10787-025-02099-w

**Published:** 2026-01-08

**Authors:** Maiara Piva, Kelly M. Yaekashi, Thais G. O. Pereira, Mariana M. Bertozzi, Felipe A. Pinho-Ribeiro, Cássia Calixto-Campos, Doumit Camilios-Neto, Sergio M. Borghi, Ana C. Zarpelon-Schutz, Victor Fattori, Rubia Casagrande, Waldiceu A. Verri

**Affiliations:** 1 Department of Immunology, Parasitology and General Pathology, Center of Biological Sciences, Londrina State University, Rod. Celso Garcia Cid Pr 445 KM 380, P.O. box 10.011, Londrina, Paraná 86057-970 Brazil; 2 Departament of Pharmaceutical Sciences, Center of Health Sciences, Londrina State University, Londrina, Paraná Brazil; 3 Division of Dermatology, Department of Medicine, Washington University School of Medicine in St. Louis, Saint Louis, MO USA; 4 Department of Biochemistry and Biotechnology, Centre of Exact Sciences, Londrina State University, Londrina, PR 86057-970 Brazil; 5 Center for Research in Health Sciences, University of Northern Paraná, Londrina, Paraná Brazil; 6 Federal University of Parana, Campus Toledo, Toledo, Paraná 85919-899 Brazil; 7 Departament of Vascular Biology Program, Department of Surgery, Boston Children’s Hospital-Harvard Medical School, Karp Research Building, Boston, MA USA

**Keywords:** Flavonoid, Analgesic, Pain, TRPV1, TRPA1, *Trans*-Chalcone, Calcium, Dorsal root ganglia neuron

## Abstract

**Objective:**

*Trans*-Chalcone (TC) is an anti-inflammatory flavonoid that reduces hyperalgesia by targeting nuclear factor κB and inflammasome in gout arthritis model. However, a direct modulation of nociceptors by TC has never been investigated, which was the aim of the present study.

**Methods:**

Experimental models of overt pain-like behaviors were applied as the stimuli-induced behavior depends, at least in part, on nociceptive neuron activation by the stimuli themselves making them suitable to investigate if a drug candidate can inhibit nociceptive neuron activation. The selected models involve transient receptor potential (TRP) vanilloid 1 (V1)^+^ and TRP ankyrin 1 (A1)^+^ nociceptive neuron activation.

**Results:**

TC (10 mg/kg, per oral, 30 min pretreatment) inhibited abdominal contortions induced by acetic acid (58.8%) and phenyl-*p*-benzoquinone (PBQ—54.6%), and paw flinching (44 and 48%) and licking (38 and 46%) triggered by formalin and complete Freund’s adjuvant (CFA—46 and 43%), indicating TC inhibits varied overt pain-like behaviors. Considering TRPV1 and TRPA1 channels are activated in those models, TC activity was also tested in experimental conditions in which capsaicin (a TRPV1 agonist)- and allyl isothiocyanate (AITC, a TRPA1 agonist)-triggered nociceptive behavior. TC inhibited capsaicin (44 and 37.5%) and AITC (35.1 and 52%) paw flinching and licking behavior. TC (3 μM) also reduced the calcium influx caused by capsaicin (30%) and AITC (37.6%) stimulation of primary dorsal root ganglia neurons. Additionally, TC inhibited CFA-induced hyperalgesia, paw inflammation without toxic effects.

**Conclusions:**

TC reduces overt pain-like behavior, at least in part, by inhibiting nociceptive neuron TRPV1 and TRPA1 channels activation.

**Supplementary Information:**

The online version contains supplementary material available at 10.1007/s10787-025-02099-w.

## Introduction

Pain is a symptom of many diseases and represents a significant public health issue because it affects about 30% of the adult population globally, and due to impairing daily activities, sleep, and interpersonal relationships (Cominelli et al. 2025; Zimmer et al. 2021; Macchia et al. 2023). Pain management relies on analgesics, which include several classes of medications, such as nonsteroidal anti-inflammatory drugs, antidepressants, antiepileptics, local anesthetics, opioids and acetaminophen (Milani and Davis 2023). Despite ongoing efforts, acute pain relief remains suboptimal due to considerable adverse effects, potential of addiction, insufficient efficacy and/or tolerance development, highlighting the need for novel analgesic options with higher safety profiles. A valuable experimental tool to screen potential analgesic drugs and to identify their mechanisms of action is the assessment of overt pain-like behavior (Pavao-De-Souza et al. 2012).

To understand overt pain-like behavior, it is imperative to differentiate sensation and perception: the sensation refers to the actual detection of noxious stimuli or nociception that can occur much faster than the brain’s perception or interpretation of that nociceptive input, which in turn is influenced by context and multiple other related physiopathological factors. Thus, pain involves not only the detection of a nociceptive input, but also its interpretation loaded with personal experiences and individual physiopathological characteristics (Prescott and Ratté 2016). The cells responsible for the detection of external stimuli sensation are first-order sensory neurons, also known as primary afferents, and they convey that information through action potentials (Prescott and Ratté 2016). It is accepted that overt pain-like behavior occurs due to the interaction of a noxious stimulus with nociceptive sensory neurons, generating an almost immediate response as the ionic influx generates nociceptive neuron depolarization, transmitting the peripheral nociceptive input towards the central nervous system (Kendroud et al. 2025). Therefore, overt pain-like behaviors can be quantitated to screen potentially novel analgesic drugs because analgesics in clinical use reduce such behaviors as well as to unveil nociceptive mechanisms (Verri et al. 2008).

Two members of the transient receptor potential (TRP) family, TRPV1 (TRP-vanilloid 1) and TRPA1 (TRP-ankyrin 1), are among the currently known ionic channels that mediate nociception (Miyamoto et al. 2009). TRPV1 is a multistate channel present on nociceptors, acting as a molecular transducer, which at negative holding potential activation allows calcium and sodium influx, thereby inducing cell depolarization (Rosenbaum and Simon 2007). Similarly, the TRPA1 ionic channel is involved in inflammatory and immune responses, as well as the conversion of physical and chemical stimuli in pain sensation (Heller and Liedtke 2006). TRPA1 is sensitive to oxidative stress (Miyake et al. 2016). Most of C fibers express TRPV1 and this is a phenotypic characteristic of unmyelinated nociceptors. Although in a lesser extent, some Aδ neurons also express TRPV1 and function as nociceptors. TRPA1 is mostly co-expressed with TRPV1 in a manner that 30–50% of TRPV1^+^ neurons co-express TRPA1. However, their co-modulation and crosstalk are less clearly understood (Story et al. 2003; De Araujo et al. 2020; Maximiano et al. 2024).

*Trans*-Chalcone (TC; C_15_H_12_O; benzylideneacetophenone) is a flavonoid precursor composed by a core structure that can be described as the backbone of flavonoids, presenting a relatively flexible and simple chemical structure (Najafian et al. 2010). TC is an important molecule because it is a precursor of most flavonoids in plants. Additionally, TC occurs naturally in several plant sources, including Licorice (*Glycyrrhiza *sp*.*), *Angelica *sp., Turmeric (*Curcuma longa*), *Passiflora *sp., Kava-Kava (*Piper methysticum*), *Aniba riparia Didymocarpus corchorijolia* and Hawthorn (*Crataegus *sp*.*) (Singh et al. 2016; Santos et al. 2024).

Prior evidence supports TC has anti-inflammatory, antiparasitic, and antineoplasic properties (Karkhaneh et al. 2016; Lamoke et al. 2011; Martinez et al. 2017a and 2017b; Miranda-Sapla et al. 2019; Batiha et al. 2019; Bortolotto et al. 2016). So far, the analgesic activity of TC has been demonstrated in a model of gout arthritis and of complete Freund adjuvant (CFA) inflammation. TC reduced mechanical hyperalgesia in a murine model of gouty arthritis. The mechanism of action was credited to the inhibition of nuclear factor κB (NF-κB) pathway activation and of NOD-like receptor protein 3 (NLRP3) inflammasome function, which are relevant molecular targets in gout arthritis (Staurengo-Ferrari et al. 2018). TC also reduces thermal hyperalgesia induced by complete Freund adjuvant (CFA) (Jabbar et al. 2024). Per oral (p.o.) treatment with TC inhibits leukocyte recruitment, pro-inflammatory transcription factors and their downstream signaling pathways, effectively inhibiting cytokine production and oxidative stress, which are pathological mechanisms also involved in pain development (Singh et al. 2016; Karkhaneh et al. 2016; Staurengo-Ferrari et al. 2018; Guazelli et al. 2023).

TC treatment induces particularly remarkable antioxidant function of reducing lipid peroxidation and H_2_O_2_ induction of glutathione related enzymes in hepatocellular carcinoma (Sikander et al. 2011). In models of inflammatory diseases, TC impairs the nicotinamide adenine dinucleotide phosphate (NADPH) oxidase activity and improves antioxidant capacity by stimulating intrinsic mechanism of defense against oxidative stress, such as nuclear factor erythroid 2–related factor 2 (Nrf2)/antioxidant responsive elements (ARE) (Martinez et al. 2017a, 2017b; Staurengo-Ferrari et al. 2018; Guazelli et al. 2023). TC is a quite singular flavonoid because this class of polyphenols are known for their chemical structures with intrinsic antioxidant effects by donating H^+^ and acceptance of electrons in the phenolic ring. However, TC has an atypical chemical structure among flavonoid since it lacks hydroxyl groups, which results in the absence of structural chemical groups with intrinsic antioxidant activity in cell free assays (Martinez et al. 2017a). Therefore, any redox regulation in vivo by TC is related to other mechanisms that are not of intrinsic antioxidant chemical structure.

Chalcone derivatives have been previously proposed as non-canonical ligands for TRPV1. In silico docking simulation data suggest that some chalcones could occupy the TRPV1 binding site without the ability of keeping an active conformation. Benso et al. (2019) applied two chalcone derivatives in their study. The chalcone derivative (E)-1-(4-bromophenyl)-3-phenylprop-2-en-1-one induced at a concentration of 10 μM an increase of intracellular calcium in HEK293 T cells transfected to express TRPV1 channels and loaded with Fluo4AM to detect calcium. However, the response triggered by the chalcone derivative was transient and quickly vanishing after an initial rise and reaching solely up to 50% of maximal burst caused by capsaicin whose response was long lasting at a smaller concentration of 1 μM. The second derivative tested, (E)-1-(4-bromophenyl)-3-(4-hydroxyphenyl) prop-2-en-1-one, did not triggered intracellular calcium rise. The joint data lead to the conclusion that some chalcone derivatives likely function as TRPV1 antagonists by simply occupying the capsaicin binding site and others may act as partial agonists causing TRPV1 desensitization (Benso et al. 2019). It is important to mention that it was not assessed if those chalcone derivatives would reduce capsaicin-triggered responses. Regarding TRPA1, a single study assessed the activity of 11 chalcone derivatives. Data demonstrated that those derivatives activate HEK-293 cells expressing TRPA1. The proposed mechanism was an activation-induced desensitization of TRPA1 leading to a reduction in mustard oil eye wiping and formalin nociceptive behavior (Moriello et al. 2016). The current evidence on the role of chalcones of targeting TRPV1 or TRPA1 is restricted to these two studies (Moriello et al. 2016; Benso et al. 2019), but none of them investigated the activity of TC. In the present study, we sought to investigate potential analgesic properties of TC in experimental models of overt pain-like behavior in mice, focusing on nociceptive neuron mechanisms that have not been investigated for TC yet. Due to the role of TRPV1 and TRPA1 in the nociceptive responses in the selected models and possible transient activation/ antagonism of TRPV1 and TRPA1 by other chalcones, the experimental design focused on potential TC effect over neuronal TRPV1 and TRPA1 activation.

## Materials and methods

### Animals

Experiments were performed in 6–7 weeks old male Swiss mice, weighing 20–30 g, obtained from the Londrina State University animal facility (Londrina, PR, Brazil). Mice had access to water and food ad lib and were kept at controlled temperature (23 °C ± 2) with 12 h light/dark cycles. Standard polypropylene cages measuring 41 × 34 × 16 cm (Insight®) were used to house groups of 6 animals per cage. Behavioral tests were carried out always between 9 a.m. and 5 p.m. and acclimatization in the testing room was prepared at least 2 h before the beginning of the experiments. The experimenters were blinded to the different groups evaluated in the study. For sample collection, mice were euthanized by terminal anesthetization with isoflurane 5%, followed by decapitation procedure.

### Experimental design

**Fig. 1:**
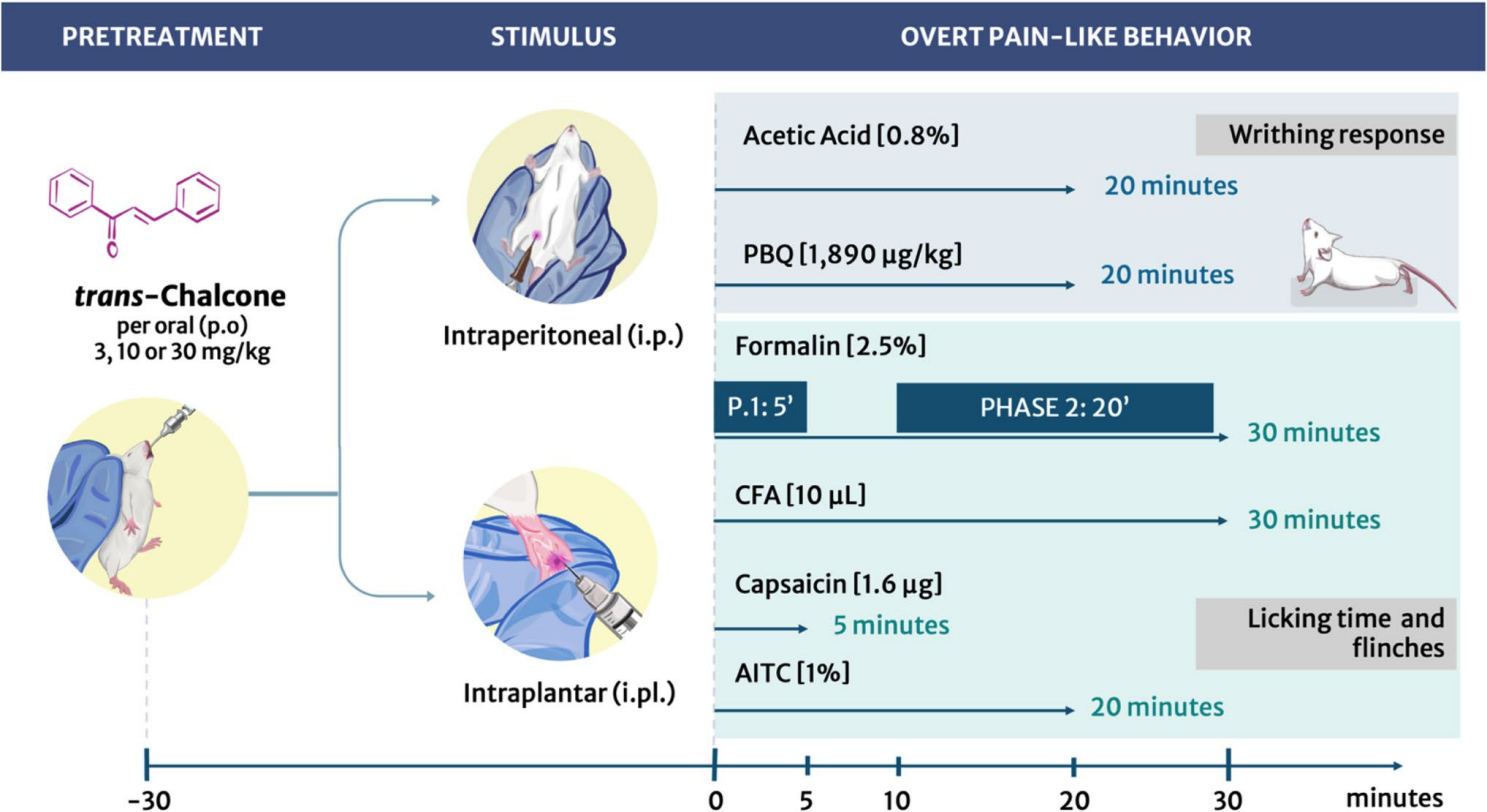
Experimental design. For behavior experiments TC treatments were administered orally by gavage 30 min before stimuli. The doses of 3, 10 and 30 mg/kg were tested initially to observe TC effects on writhing response upon acetic acid (0.8% in saline solution/200 μL per abdominal cavity) stimulus, in which the 10 mg/kg dose was chosen for the following experiments. Vehicle was prepared at the same time and equal volume as treatment, but without adding TC. The writhing response to acetic acid and PBQ were assessed for 20 min after i.p. administration of the respective stimulus (Verri et al. 2008). For the formalin test, the time spent licking the stimulated paw and number of paw flinches were separate into two phases, the first 5 min accounted for phase 1, and from the 10th minute until the 30th minute after formalin i.pl. injection was the duration of phase 2 (Pavao-de-Souza et al. 2012). The nociceptive response induced by CFA was quantitated during 30 min (Fattori et al. 2019). Capsaicin response was assessed for 5 min and AITC response for 20 min (Fattori et al. 2022)

Figure 1 presents the experimental design for in vivo assays of the present study. Mice were treated p.o. with 3, 10 or 30 mg/kg of TC (95%, Santa Cruz Biotechnology, cat # 614-47-1, Dallas, TX, USA) diluted in saline 20% tween-80 or vehicle (20% tween-80 in saline solution) 30 min before i.p. or i.pl. injections of the different inflammatory stimulus, including acetic acid (0.8%, i.p.), PBQ (1890 μg/kg, i.p.), formalin (2.5%, i.pl.), CFA (10 μl/paw), capsaicin (1.6 μg, i.pl.), and AITC (1%, i.pl.). The doses and route of administration of TC were based on previous studies of our group (Staurengo-Ferrari et al. 2018). Overt pain-like behavior tests using acetic acid and PBQ-induced abdominal writhing as well as formalin, CFA, capsaicin, and AITC-induced paw flinching and licking as stimuli were applied to evaluate TC potential analgesic effects (Verri et al. 2008; Fattori et al. 2019; Fattori et al. 2022). Confocal microscopy calcium dynamics imaging in cultured DRG neurons of naïve mice was also used to evaluate whether TC would modulate TRPV1 and TRPA1 activity (Fattori et al. 2022). In addition to overt pain-like behaviors, mechanical hyperalgesia, thermal hyperalgesia, and edema were evaluated daily 30 min after TC p.o. treatment. At the 7th day post-CFA injection, paw skin was collected and assessed for myeloperoxidase (MPO) and N-acetylglucosaminidase (NAG) activities as indirect measures of neutrophil/macrophage recruitment (Fattori et al. 2019). Blood samples were also collected for aspartate aminotransferase (AST) and alanine transaminase (ALT) levels quantitation to assess potential liver toxicity (Staurengo-Ferrari et al. 2018), and stomach samples for MPO activity to investigate ulcer formation (Wallace et al. 1995).

### Overt pain-like behavior tests

#### Writhing response

The acetic acid- and PBQ-induced writhing models were performed to investigate the analgesic properties of TC (Koster et al. 1959; Collier et al. 1968; Emele and Shanaman 1967). Acetic acid (0.8%, v/v in saline), PBQ (dimethylsulfoxide; DMSO 2%, v/v in saline, 1,890 μg/kg), or vehicle were injected into the peritoneal cavities of mice 30 min after pretreatment with TC (3–30 mg/kg, p.o.). The intensity of overt pain-like behavior is expressed as the cumulative writhing score over 20 min after stimulus.

#### Formalin test

The Formalin Test was previously described by Dubuisson and Dennin (1977). The number of paw flinches and time licking the paw were determined between 0–30 min after 25 μL formalin i.pl. injection. The period was divided into intervals of 5 min, defining the first (0–5 min) and second (15–30 min) phases, which are characteristic of the neurogenic and inflammatory phases of the test, respectively. Results are presented as the number of flinches in the first and second phases.

### CFA-induced overt pain-like behavior

Overt-pain-like behavior was determined by the number of paw flinches and time spent licking the stimulated hind paw, analyzed for 30 min after intraplantar injection with 10 μL of CFA. These behaviors are considered indicative of nociception. Results were expressed by the total number of flinches and time spent licking over 30 min (Martinez et al. 2016).

### Capsaicin- and AITC-induced overt pain-like behavior

TC (10 mg/kg, p.o.) was administered 30 min before the nociceptive stimuli injection. Paw flinching and licking were evaluated over 5 min after i.pl. injection of capsaicin (1.6 µg/25 µL/paw), or 30 min after i.pl. injection of AITC (1%, 20 µL/paw) (Fattori et al. 2022; Borghi et al. 2013). Results are expressed as the total number of flinches or time spent licking the paw (in seconds).

### Calcium influx imaging by confocal microscopy

**Fig. 2:**
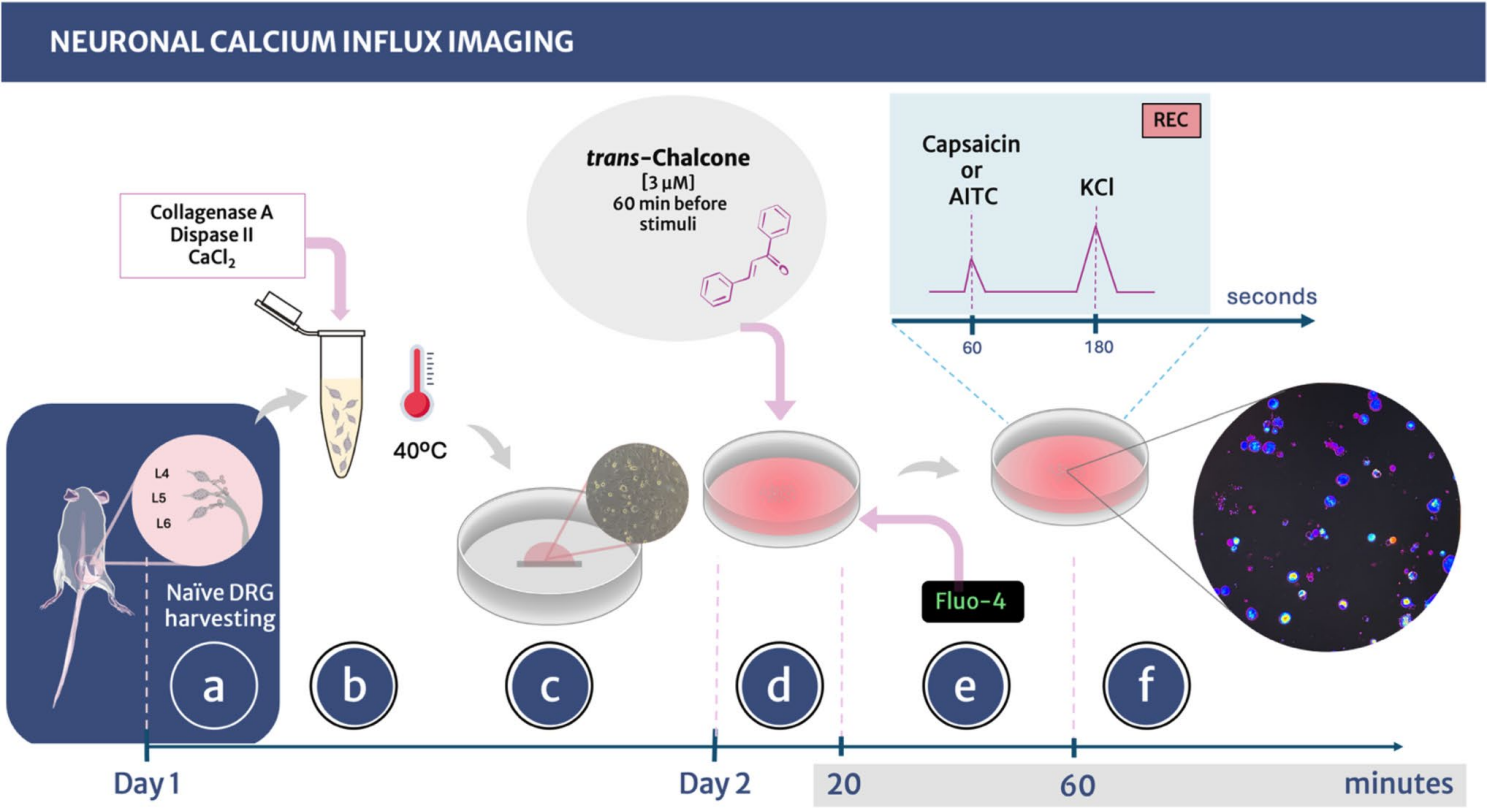
Schematic representation of neuronal calcium influx. **a** DRG from both sides of L4, L5 and L6 were collected from naïve mice. **b** Neuron somas were separated adding collagenase A, dispase II and the co-factor CaCl_2_ and kept at 40 °C to carry out enzyme digestion of the DRG extracellular matrix. **c** Neuron somas were seeded on culture dishes previously coated with laminin, neurobasal medium was added and cells were incubated overnight at 37 °C and 5% CO_2_ for stabilization. **d** In the next day, the medium was removed, cells were washed twice with HBSS to remove unattached cells, and fresh medium was added with TC or vehicle one hour prior to the assay. **e** Fluo-4 was added to the culture medium and incubated for 40 min. **f** Plates were washed twice with HBSS Ca^++^Mg^++^ to remove the remaining treatments and fluorescent probe, and cultures were recorded for 4 min. At the 60 s-mark, Capsaicin or AITC was added, and at the 180 s-mark, KCl was added followed by additional 60 s recording

Naïve dorsal root ganglia (DRG) neurons were harvested bilaterally between L_4_-L_6_ vertebrae (Fig. 2a**)** into culture medium with Dulbecco’s modified Eagle medium (DMEM) high glucose 10% fetal bovine serum (FBS), enzymatically disrupted by adding and collagenase A/dispase II solution, supplemented with 5 mM CaCl_2_ (Collagenase A, Roche cat # 10103578001, Dispase II, Roche cat # 04942078001) incubation at 40 °C, and mechanically disrupted by gentil pipetting until complete dissociation (Fig. 2b). DRG cells were then centrifuged (200× g for 5 min) to obtain a monolayer of DRG axotomized neurons placed over a glass-bottom culture plate pre-coated with laminin (Fig. 2c). Cells were kept at 37 °C, 5% CO_2_ overnight in neurobasal medium (NBM; Invitrogen cat # 21103-049) and supplemented with 2% B27 serum-free supplement (50x, Invitrogen cat # 17504-044) 1% Pen/Strep (Cellgro cat # 15140-163), 1% L-Glutamine (Invitrogen cat # 25030-164), 0.004% ARA-C 0.004% glial-derived neurotrophic factor (GDNF) and 0.002% nerve growth factor (NGF). Culture plates were seeded by duplicates and pretreated with TC (3 μM) or vehicle (0,01% DMSO in Hanks’ balanced salt solution (HBSS) for one hour before stimuli (Fig. 2d). This concentration of TC was selected based on prior data demonstrating it is active in macrophages (Staurengo-Ferrari et al. 2018). Cells were then loaded with 1.2 μM of Fluo‐4AM probe for 40 min (Fig. 2e) (same concentrations for all experimental conditions), plates were washed, and the medium was replaced by HBSS with calcium and magnesium for neuron stimuli imaging in a confocal microscope (TCS SP8, Leica Microsystems). To assess TRPV1 and TRPA1 activation, DRG plates were recorded for 4 min, divided into 1 min of initial reading (0‐s mark, baseline values), followed by stimulation with capsaicin (1 μM, TRPV1 agonist) or AITC (100 nM, TRPA1 agonist) for 2 min at the 60‐seconds mark and KCl (40 mM, a viability control for cells) for 1 min at the 180‐s mark (Fig. 2f). Calcium influx was analyzed based on the mean fluorescence intensity detected in each neuron with the LAS X Software along the time course (Leica Microsystems). Despite Fluo-4’s green fluorescence range (Ex/Em of Ca^2+^–bound form 494/506 nm), by using the color scale different levels of fluorescence intensity are converted into the observed color spectrum, where purple/blue represent low fluorescence and red is the maximum fluorescence intensity. Experiment recordings were obtained using the 20× objective lens.

### Mechanical hyperalgesia, thermal hyperalgesia, and paw edema

Following CFA (10 μL) paw stimulus, mechanical and thermal hyperalgesia, and edema were evaluated daily (Borghi et al. 2013). Mechanical hyperalgesia was measured with the aid of an electronic von Frey apparatus (Insight, Ribeirao Preto, Brazil). Mice were placed over a wired grid and uprising pressure was slowly applied to the center of the right hind paw with the aid of a polypropylene tip attached to the electronic von Fray apparatus until paw withdrawal. Results are presented as the delta of applied the force (Δg), obtained by the difference in force applied before and after stimuli. Thermal hyperalgesia was assessed placing each mouse individually over a hot plate (Insight, Ribeirao Preto, Brazil) set at 52 °C ± 1 for 20 s or until the display of nociceptive behaviors such as flinting or licking a hind paw. Results are presented as latency (in seconds). Edema formation was measured with an analogic micrometer (Mitutoyo, Kawasaki, Japan) and is displayed as Δmm.

### MPO and NAG activity assessment

MPO (paw skin and stomach) and NAG (paw skin) activities (Ferraz et al. 2021) were quantitated 7 days after CFA i.pl. injection and TC daily (10 mg/kg, p.o.) treatment. For the MPO assay, tissue samples were collected in 50 mM K_2_PO_4_ buffer (pH 6.0) containing 0.5% HTAB and were mechanically disrupted. Homogenates were centrifuged (16.000× g, 10 min, 4 °C) and 10 μL of the supernatant were added to 200 μL of 50 mM phosphate buffer, pH 6.0, containing 0.167 mg/mL o-dianisidine dihydrochloride and 0.015% hydrogen peroxide to determine MPO activity. Samples optical densities were measured at 450 nm (Multiskan GOMicroplate Spectrophotometer, ThermoScientific, Finland) and compared to a standard curve made with known neutrophils concentrations. To assess NAG activity, 20 μL of the supernatant were added to 80 μL of 50 mM phosphate buffer (pH 6.0), 2.24 mM 4-nithophenyl N-acetyl-β-D-glucosaminidase and 100 μL of 0.2 M glycine buffer (pH 10.6). O.D. were obtained at 400 nm. Results were interpolated to a standard curve made with known macrophages concentrations.

### AST and ALT levels

Liver lesion was assessed by AST and ALT levels were quantitated in blood samples collected after 7 days of CFA i.pl. injection and TC daily (10 mg/kg, p. o.) treatment. Plasma was obtained from whole blood collected with heparin and subjected to centrifugation (200× g, 10 min, 4 °C). Samples were processed according to manufacturer’s instructions (Labtest, Lagoa Santa, Brazil) and results are presented as U/L of plasmatic AST or ALT.

### Statistical analysis

Results are presented as means ± standard error means (SEM) of measurements of 6 mice per group per experiment and are representative of two separate experiments. Sample size was determined using G*Power 3.1 (Faul et al. 2009) through repeated-measures analysis of variance (ANOVA), within between interaction and effect size f = 0.7, α probability error = 0.05, power of analysis (1-β probability error = 0.95), correlation between repeated measures = 0.5, and nonsphericity correction ε = 1. These denominators were chosen based on previous study of our group (Verri et al. 2008; Fattori et al. 2019 and 2022). The method of block randomization was used to randomize subjects into groups which results in equal sample sizes at all time points for each assay. Data with three or more groups were submitted to Shapiro–Wilk normality test and Brown-Forsythe homogeneity of variance test before proceeding to parametric or non-parametric analysis, whereas data with *p*-value ≥ 0.05 for both tests were statistically analyzed by one- or two-way ANOVA followed by Tukey’s test for multiple comparisons. Comparisons between two groups with normal distribution were conducted by t test, and group variance by f test. Statistical analyses were performed with GraphPad Prism 9 software (GraphPad Software Inc., San Diego, CA, USA). Results were considered significantly different if *p*-value ≤ 0.05. Table 1 and table S1 present a summary of statistical analysis.

**Table 1:** Summary of statistical analyzes

Acetic Acid and PBQ overt pain-like behavior			Normality		Equality of means		Statistical test		Post-test	
Result			Shapiro–Wilk test		Brown-Forsythe test		One-way ANOVA		Tukey’s multiple comparisons test	
	Groups	n	W	*P value*	F (DFn, DFd)	*P value*	F (DFn, DFd)	*P value*	Comparison	*P value*
Figure 3b	Saline	6	0.8268	0.101	2.522 (4, 25)	0.0664	67.59 (4, 25)	< 0.0001	Saline versus 0	< 0.0001
	0	6	0.9607	0.8253					0 versus 3	0.1437
	3	6	0.9301	0.5812					0 versus 10	< 0.0001
	10	6	0.982	0.5647					0 versus 30	< 0.0001
	30	6	0.9182	0.4928					10 versus 30	0.7803
Figure 3c	Saline	6	0.8216	0.0911	2.669 (2, 15)	0.102	85.90 (2, 15)	< 0.0001	Saline versus 0	< 0.0001
	0	6	0.9005	0.3772					Saline versus 10	< 0.0001
	10	6	0.8907	0.3218					0 versus 10	< 0.0001

Formalin overt pain-like behavior			Normality		Statistical test		Post-test			
Result			Shapiro–Wilk test		Two-way ANOVA		Tukey’s multiple comparisons test			
	Flinches	n	W	*P *value		*P* value	Comparison	*P *value	Comparison	*P *value
Figure 4b					Time × Column Factor	0.236	0–5 min		10–30 min	
	0–5 min	6	0.9994	0.9516	Time	0.0361	Saline versus 0	< 0.0001	Saline versus 0	< 0.0001
	10–30 min	6	0.9516	0.8006	Column Factor	< 0.0001	Saline versus 10	0.0023	Saline versus 10	< 0.0001
					Subject	0.7933	0 versus 10	0.0051	0 versus 10	0.0011

Formalin overt pain-like behavior			Normality		Statistical test		Post-test			
Result			Shapiro–Wilk test		Two-way ANOVA		Tukey’s multiple comparisons test			
	Licking time	n	W	*P* value		*P *value	Comparison	*P *value	Comparison	*P *value
Figure 4c					Time × Column Factor	0.0131	0–5 min		10–30 min	
	0–5 min	6	0.9949	0.8633	Time	0.0005	Saline versus 0	< 0.0001	Saline versus 0	< 0.0001
	10–30 min	6	0.9969	0.8934	Column Factor	< 0.0001	Saline versus 10	0.0064	Saline versus 10	< 0.0001
					Subject	0.286	0 versus 10	0.0379	0 versus 10	0.0002

CFA overt pain-like behavior			Normality		Equality of group variances		Statistical test		Post-test	
Result			Shapiro–Wilk test		Brown-Forsythe test		One-way ANOVA		Tukey’s multiple comparisons test	
	Group	n	W	*P *value	F (DFn, DFd)	*P *value	F (DFn, DFd)	*P *value	Comparison	*P *value
Figure 5b	Saline	6	0.8139	0.078	2.197 (2, 15)	0.1457	43.43 (2, 15)	< 0.0001	Saline versus 0	< 0.0001
	0	6	0.8641	0.2039					Saline versus 10	0.0006
	10	6	0.9037	0.3963					0 versus 10	0.0012
Figure 5c	Saline	6	0.875	0.2468	2.750 (2, 15)	0.0961	46.66 (2, 15)	< 0.0001	Saline versus 0	< 0.0001
	0	6	0.9155	0.4739					Saline versus 10	0.0002
	10	6	0.9167	0.4819					0 versus 10	0.0021

Capsaicin and AITC overt pain-like behavior			Normality		Equality of group variances		Statistical test		Post-test	
Result			Shapiro–Wilk test		Brown-Forsythe test		One-way ANOVA		Tukey’s multiple comparisons test	
	Group	n	W	*P *value	F (DFn, DFd)	*P *value	F (DFn, DFd)	*P* value	Comparison	*P *value
Figure 6b	Saline	6	0.824	0.0956	1.646 (2, 15)	0.2258	34.97 (2, 15)	< 0.0001	Saline versus 0	< 0.0001
	0	6	0.9441	0.692					Saline versus 10	0.0004
	10	6	0.8716	0.2327					0 versus 10	0.0151
Figure 6c	Saline	6	0.8232	0.0941	0.5339 (2, 15)	0.5971	64.27 (2, 15)	< 0.0001	Saline versus 0	< 0.0001
	0	6	0.8916	0.3264					Saline versus 10	< 0.0001
	10	6	0.8862	0.2988					0 versus 10	0.0002
Figure 6d	Saline	6	0.9006	0.3778	3.607 (2, 15)	0.0526	38.63 (2, 15)	< 0.0001	Saline versus 0	< 0.0001
	0	6	0.8955	0.3481					Saline versus 10	0.005
	10	6	0.7963	0.0544					0 versus 10	0.0004
Figure 6e	Saline	6	0.9076	0.4207	2.692 (2, 15)	0.1003	75.78 (2, 15)	< 0.0001	Saline versus 0	< 0.0001
	0	6	0.91	0.4362					Saline versus 10	< 0.0001
	10	6	0.8241	0.0957					0 versus 10	0.0018

Calcium Influx—Capsaicin			Shapiro–Wilk test		f test		Unpaired t test	
	Group	n (plates)	W	*P *value	F (DFn, Dfd)	*P* value	Comparison	*P *value
Figure 7d	Vehicle	4	0.9448	0.6839	8.794 (3, 3)	0.1073	TC versus Vehicle	0.0194
	TC	4	0.8313	0.1711				

Calcium Influx – AITC			Shapiro–Wilk test		f test		Unpaired t test	
	Group	n (plates)	W	*P *value	F (DFn, Dfd)	*P* value	Comparison	*P *value
Figure 8d	Vehicle	4	0.9292	0.5896	1.165 (3, 3)	0.9029	TC versus Vehicle	< 0.0001
	TC	4	0.8945	0.4042				

n = sample size

*P *value = probability value

W = normality computed by Shapiro-Wilk test

F = ratio of two components of the variance

DFn = degrees of freedom for numerator

DFd = degrees of freedom for denominator

Dfd = degrees of freedom for denominator (for the original f test)

## Results

### TC inhibits acetic acid- and phenyl-p-benzoquinone (PBQ)- induced overt pain-like behavior

**Fig. 3:**
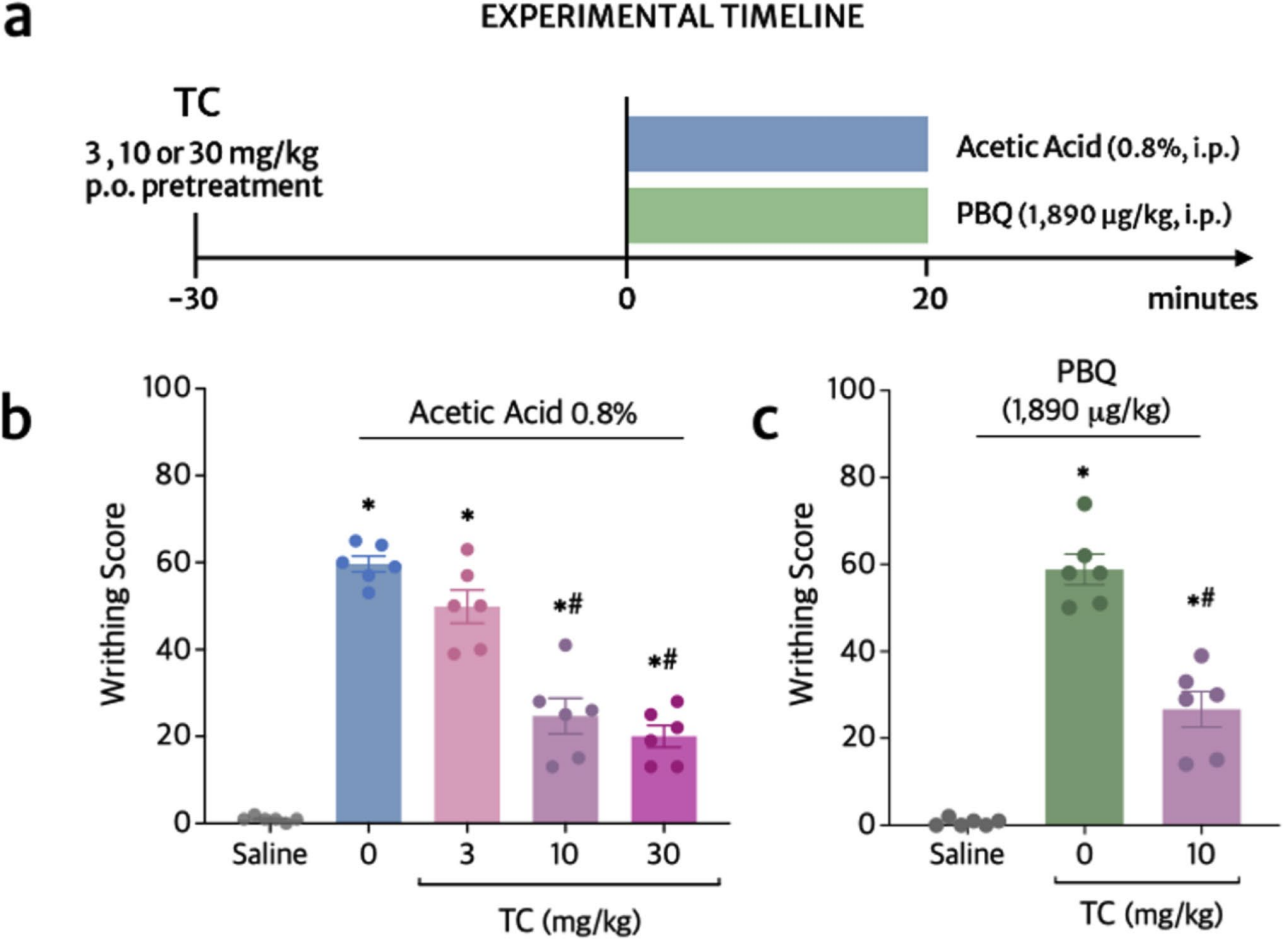
TC inhibits acetic acid- and PBQ-induced abdominal writhing. **a** Timeline for writhing response assays. Mice were treated with TC (3–30 mg/kg) or vehicle (saline 20% Tween-80) 30 min before acetic acid (**b**), or PBQ (**c**) intraperitoneal (i.p.) stimuli. The vehicle of acetic acid was saline and the vehicle of PBQ was 2% DMSO in saline. The cumulative number of abdominal contortions (writhing score) was evaluated over 20 min for both assays. Results are presented as means ± SEM of six mice per group. Shapiro–Wilk and Brown-Forsythe tests were used to assess normality and equality of group variances, respectively. Statistical analyses were performed by one-way ANOVA followed by Tukey’s multiple comparison test (Table 1). *p ≤ 0.05 compared to saline group; ^#^*p *≤ 0.05 compared to stimulus group

Acetic acid and PBQ are stimuli used to induce abdominal writhing in drug screening and physiopathological studies (Verri et al. 2008). Initially, we investigated the analgesic effects of TC (3–30 mg/kg) on overt pain-like behavior induced by acetic acid (0.8%) and determined the optimal dose of the flavonoid for the following experiments (Fig. 3a). The dose–response indicated that the lower dose of TC (3 mg/kg) did not induce a significant reduction of acetic acid-induced abdominal contortions (16.6% inhibition compared to vehicle group) (Fig. 3a). On the other hand, the doses of 10 and 30 mg/kg had similar significant inhibitory effects on the number of abdominal contortions, of 58.8% and 66.5%, respectively. The activity of the dose of 30 mg/kg of TC started at 6 min, which was earlier than what was observed for the dose of 10 mg/kg (8 min). Considering that no significant difference was statistically detected between the doses of 10 and 30 mg/kg of TC (*p*-value = 0.7803) (Fig. 3b), the dose of 10 mg/kg was chosen for the following experiments. The IC_50_ calculated based in Fig. 3b was ~ 10.1 mg/kg of TC. This selected dose was also efficient in significantly inhibiting the writhing response induced by PBQ when compared to vehicle-treated mice (54.6% inhibition) (Fig. 3c).

### TC inhibits formalin-induced overt pain-like behavior

**Fig. 4:**
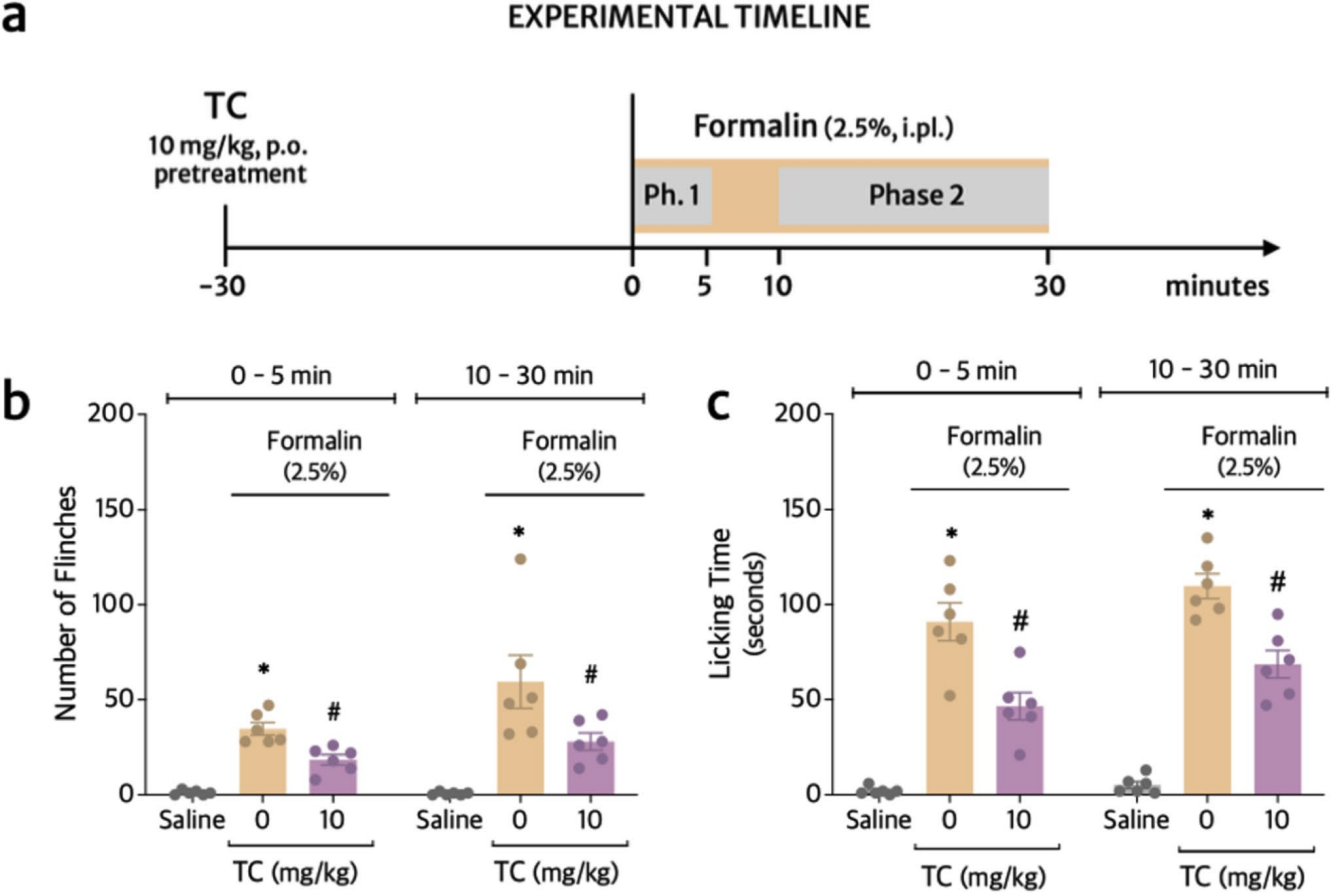
TC inhibits formalin-induced paw flinching and licking. **a** Experimental timeline. Mice were treated orally with vehicle (20% Tween-80 Saline) or TC (10 mg/kg) 30 min before the stimulus with formalin into the right hind paw. The total number of flinches (**b**) and total paw licking time (**c**) were initially evaluated for 5 min (0–5) at Phase 1, then for 20 min (10–30) at Phase 2 after the injection of formalin. Results are presented as means ± SEM of six mice per group. Shapiro–Wilk test was used to assess normality, and statistical analyses were performed by Two-way ANOVA followed by Tukey’s multiple comparison test (Table 1). *p ≤ 0.05 compared to saline group; ^#^*p *≤ 0.05 compared to stimulus group

After stablishing the best dose of TC for the present experimental condition (Fig. 4a), the formalin test was performed. As it can be observed in Fig. 4b and c, the dose of 10 mg/kg of TC significantly inhibited the first (44%) and second phases (48%) of formalin-induced paw flinches (Fig. 2B), as well as the first (38%) and second phases (46%) of formalin-induced paw licking time (Fig. 4c), suggesting TC can target the transient depolarization and excitation of primary afferent neurons and later inflammatory response.

### TC inhibits CFA-induced overt pain-like behavior

**Fig. 5:**
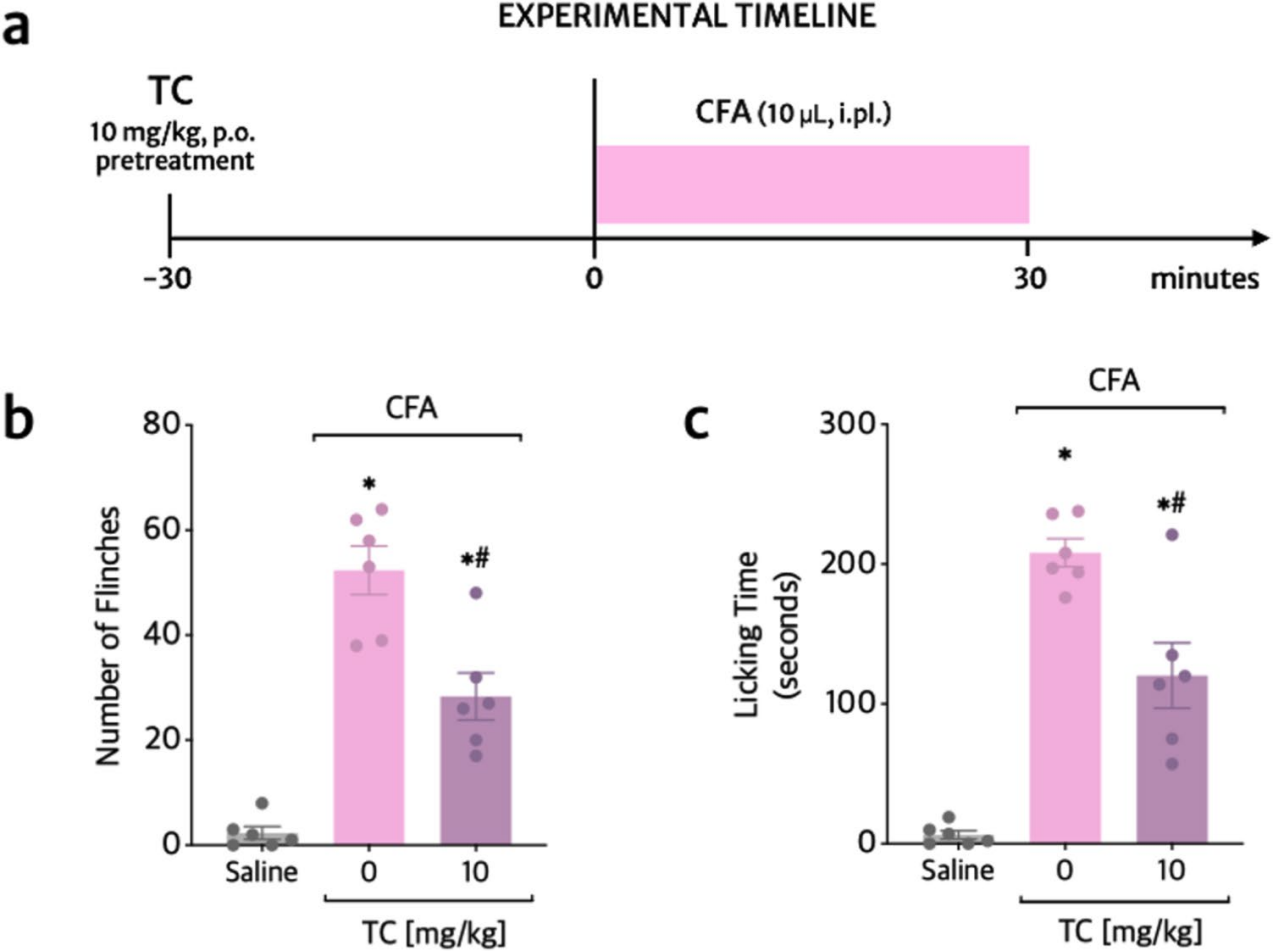
TC inhibits CFA-induced paw flinching and licking time. **a** Experimental timeline. Mice were treated orally with vehicle (20% Tween-80 Saline) or TC (10 mg/kg) 30 min before the stimulus with CFA. The total number of flinches (**b**) and total paw licking time (in seconds) (**c**) were evaluated for 30 min after CFA stimulus. Results are presented as means ± SEM of six mice per group. Shapiro–Wilk and Brown-Forsythe tests were used to assess normality and equality of group variances, respectively. Statistical analyses were performed by one-way ANOVA followed by Tukey’s multiple comparison test (Table 1). *p ≤ 0.05 compared to saline group; ^#^*p* ≤ 0.05 compared to stimulus group

The following nociceptive test was triggered by the complete Freund’s adjuvant (CFA) (Fig. 5a). TC inhibited the CFA triggered paw flinches (46%-Fig. 5b) and the time spent licking the paw (43%-Fig. 5c) during the 30 min of assessment following CFA-injection.

### TC inhibits capsaicin- and AITC-induced overt pain-like behavior

**Fig. 6:**
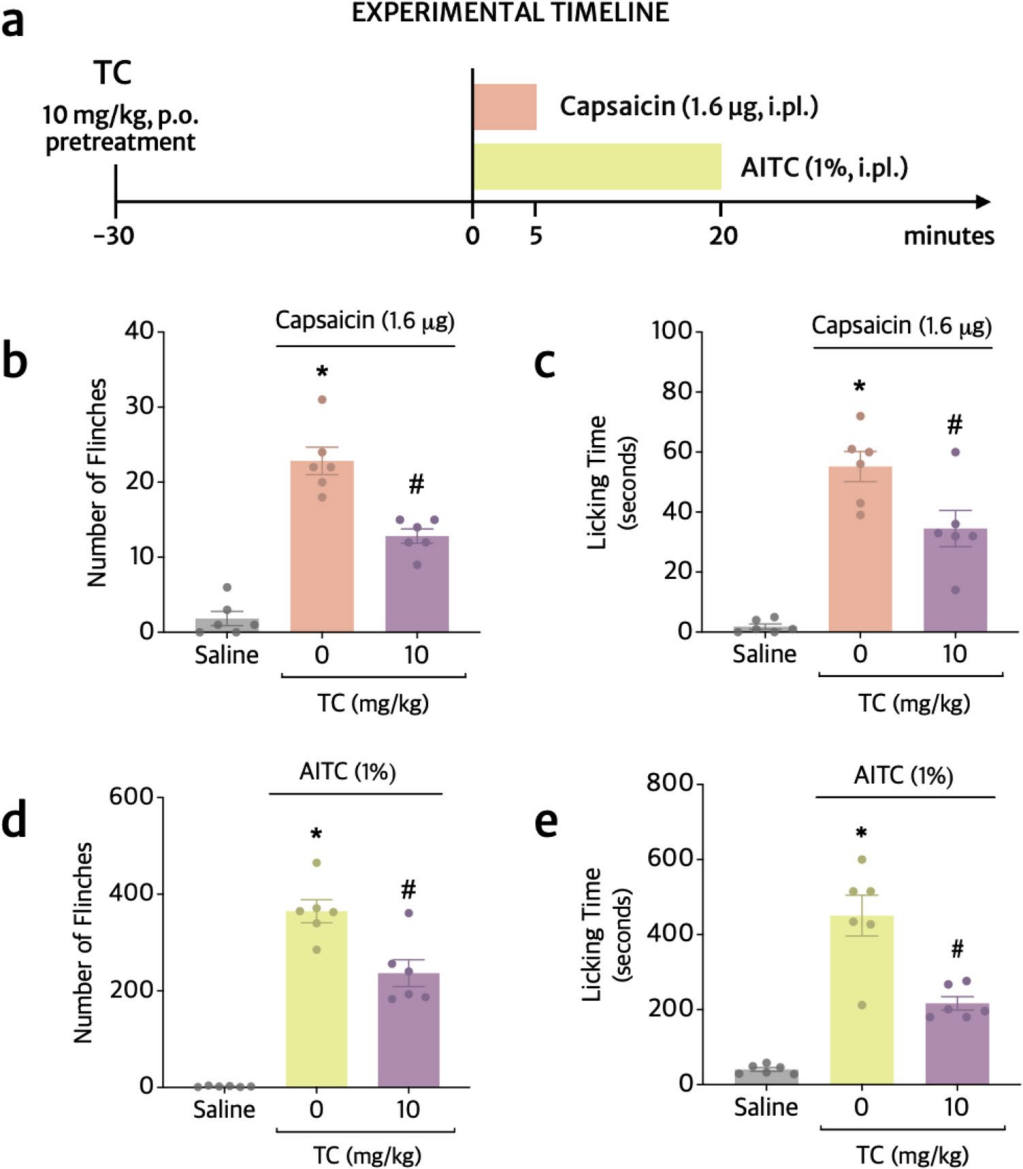
TC inhibits capsaicin (TRPV1 agonist)- and AITC (TRPA1 agonist)-induced overt pain-like behavior. **a** Experimental timeline. Time spent licking the stimulated paw (**b**) and total number of paw flinches (**c**) were counted during 5 min after i.pl. injection of capsaicin (1.6 μg/25 μL/paw). The time spent licking the stimulated paw (**d**) and total number of paw flinches (**e**) were counted during 20 min after AITC (1% v/v [10 M] in 20 μL). Results are presented as means ± SEM of six mice per group. Shapiro–Wilk and Brown–Forsythe tests were used to assess normality and equality of group variances, respectively. Statistical analyses were performed by one-way ANOVA followed by Tukey’s multiple comparison test (Table 1). *p ≤ 0.05 compared to saline group; ^#^*p* ≤ 0.05 compared to stimulus group

The acetic acid writhing response depends at least in part on TRPV1 activation (Liu et al. 2013). The CFA nociception model also depends on TRPV1 (Kanai et al. 2007) while the formalin nociceptive response depends on TRPA1 (McNamara et al. 2007). Therefore, we reason that the explanation underlying the TC analgesia could involve the inhibition of TRPV1 and TRPA1 activation. To assess whether TC effects is related to the inhibition of TRPV1 and/or TRPA1 ionic channels, in the next set of experiments, agonists of TRPV1 (capsaicin) and TRPA1 (agonist allyl isothiocyanate, AITC) were used to trigger overt pain-like behavior (Fig. 6a). The results demonstrate that TC significantly inhibited capsaicin-induced flinches in 44% (Fig. 6b) and licking time in 37.5% (Fig. 6c) during the 5 min of assessment. Likewise, the number of flinches and time spent licking the paw after the injection of AITC were inhibited in 35.1% (Fig. 6d) and 52% (Fig. 6e), respectively.

### TC reduces the activation of TRPV1^+^ and TRPA1^+^ dorsal root ganglia (DRG) neurons

For in vitro studies, we selected a concentration of TC based on Staurengo-Ferrari et al. (2018) that applied two treatment protocols to mimic the main pathological mechanism of gout arthritis in bone-marrow derived macrophages. In this system there is signal 1 that can be gram-negative bacteria lipopolysaccharide (LPS). This signal induces NFkB activation and the expression of NLRP3 (nucleotide-binding domain, leucine-rich–containing family, pyrin domain–containing-3) inflammasome components (NLRP3, ASC [apoptosis-associated speck-like protein containing a CARD], pro-caspase-1, pro-IL-1β and pro-IL-18). Then, there is signal 2, the monosodium urate crystals that will be phagocyted, cause the rupture of the phagolysosome and cathepsins will be sensed by NLRP3 causing the assembling and activation of this molecular platform to maturate IL-1β and IL-18 (Staurengo-Ferrari et al. 2018). TC treatment before signal 1 presented an IC50 of ~ 1.3 μM. When TC was used as a post-signal 1 and pre-signal 2 the IC50 was of ~ 3.1 μM. When the treatment was pre-signal 1, the concentration of 3 μM achieved maximal inhibition with a progressive response, indicating it is not supramaximal concentration. When TC treatment occurred after signal 1 and before signal 2, the concentration of 3 μM reached nearly 50% inhibition. Therefore, we reason that 3 μM would be an ideal concentration because depending on the experimental setting it would achieve between 50–100% inhibition and would not be a supramaximal concentration (Staurengo-Ferrari et al. 2018).

Furthermore, before assessing whether TC would inhibit capsaicin and AITC neuronal activation, we tested if TC per se would induce calcium influx in DRG neurons in basal conditions (Fig. S1). Fig. S1a shows DRG neuronal culture representative images. Fig. S1b presents the percentage of neuronal activation. Fig. S1c presents the basal calcium levels during 60 s. Then, the calcium levels following 120 s (from 6-s to 180 s) of TC or vehicle addition to the neuronal culture, and finally the KCl stimulation to detect the viable neurons. Fig. S1d presents the comparison of calcium levels between TC and vehicle. These results demonstrate that TC per se cannot activate DRG neurons at the concentration of 3 μM (Fig. S1). If TC per se was capable of activating TRPV1 and TRPA1 channels, it would be expected that calcium levels would rise, but this was not the case. Therefore, TC seems to be different of other chalcones that induce TRPA1 activation followed by its desensitization (Moriello et al. 2016).

**Fig. 7:**
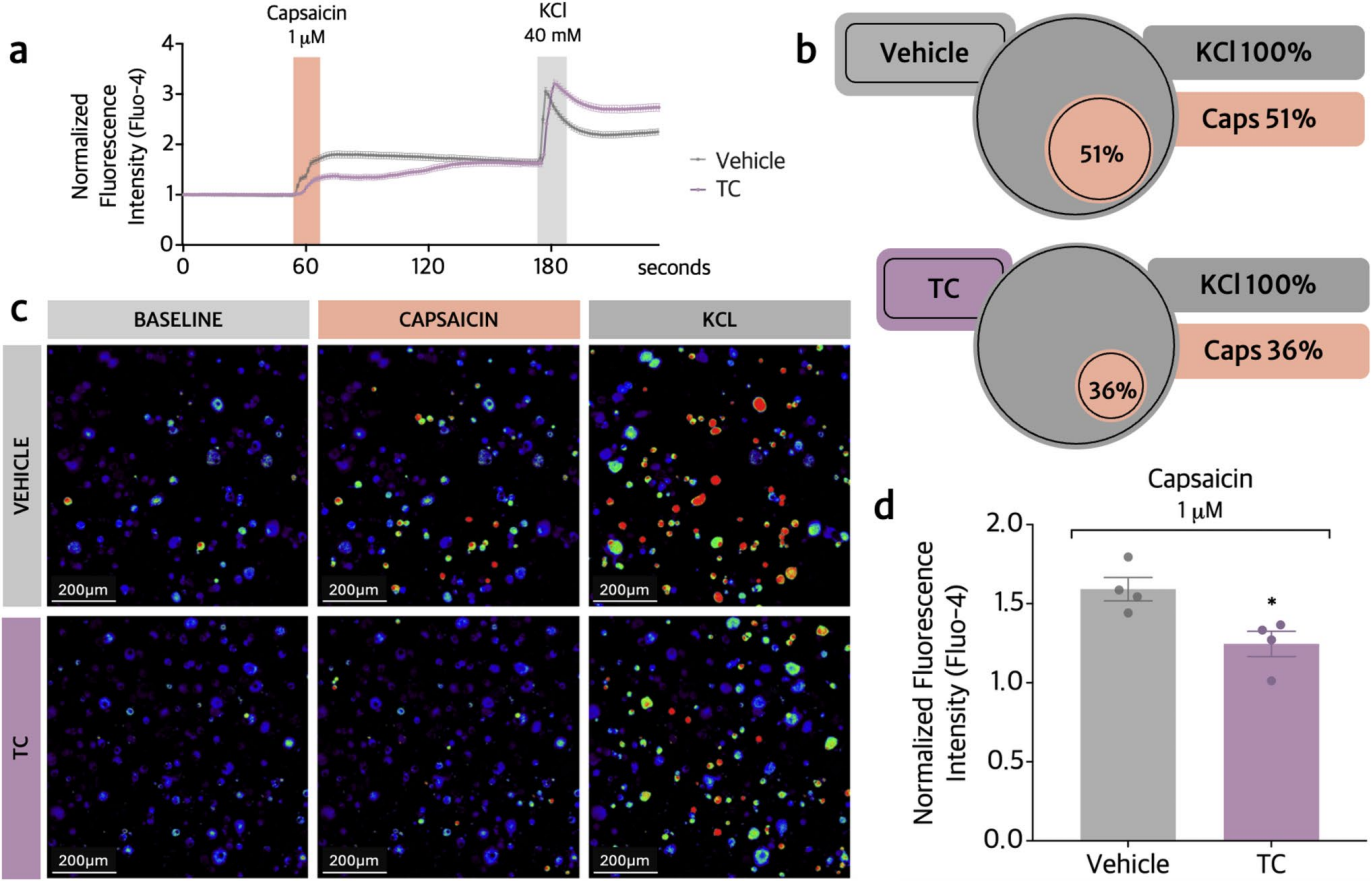
TC inhibits TRPV1^+^ nociceptive neuron activation in vitro. To assess TRPV1^+^ neuron activation in vitro, plates containing naïve DRG neurons were pretreated with TC (3 μM) or vehicle (HBSS 0.01% DMSO) for 60 min. Plates were recorded for 4 min: 1 min of initial reading, followed by stimulus with capsaicin (1 μM in HBSS) for 2 min at the 60 s-mark, and with KCl for 1 min at the 180 s-mark (40 mM, activates all neurons). **a** Normalized fluorescence intensity tracers throughout the 4 min of recording. **b** Venn’s diagram indicating the percentage of DRG-derived neurons with increased calcium influx after capsaicin stimulus. **c** Representative images acquired at baseline, Capsaicin stimulus, and KCl activation, for TC and vehicle groups. Captured with 20× objective lens. Scale bars = 200 μm **d** Comparative mean fluorescence intensity between vehicle and TC groups after capsaicin stimulus. Results are provided as means ± SEM of four culture dishes per group and each culture dish was a pool of DRG from 5 mice (n = 4 culture dishes). Vehicle group = total of 360 neurons; TC group = total of 399 neurons. Statistical analyses were performed by Shapiro–Wilk, and unpaired t test (Table 1). *p ≤ 0.05 compared to vehicle group

**Fig. 8:**
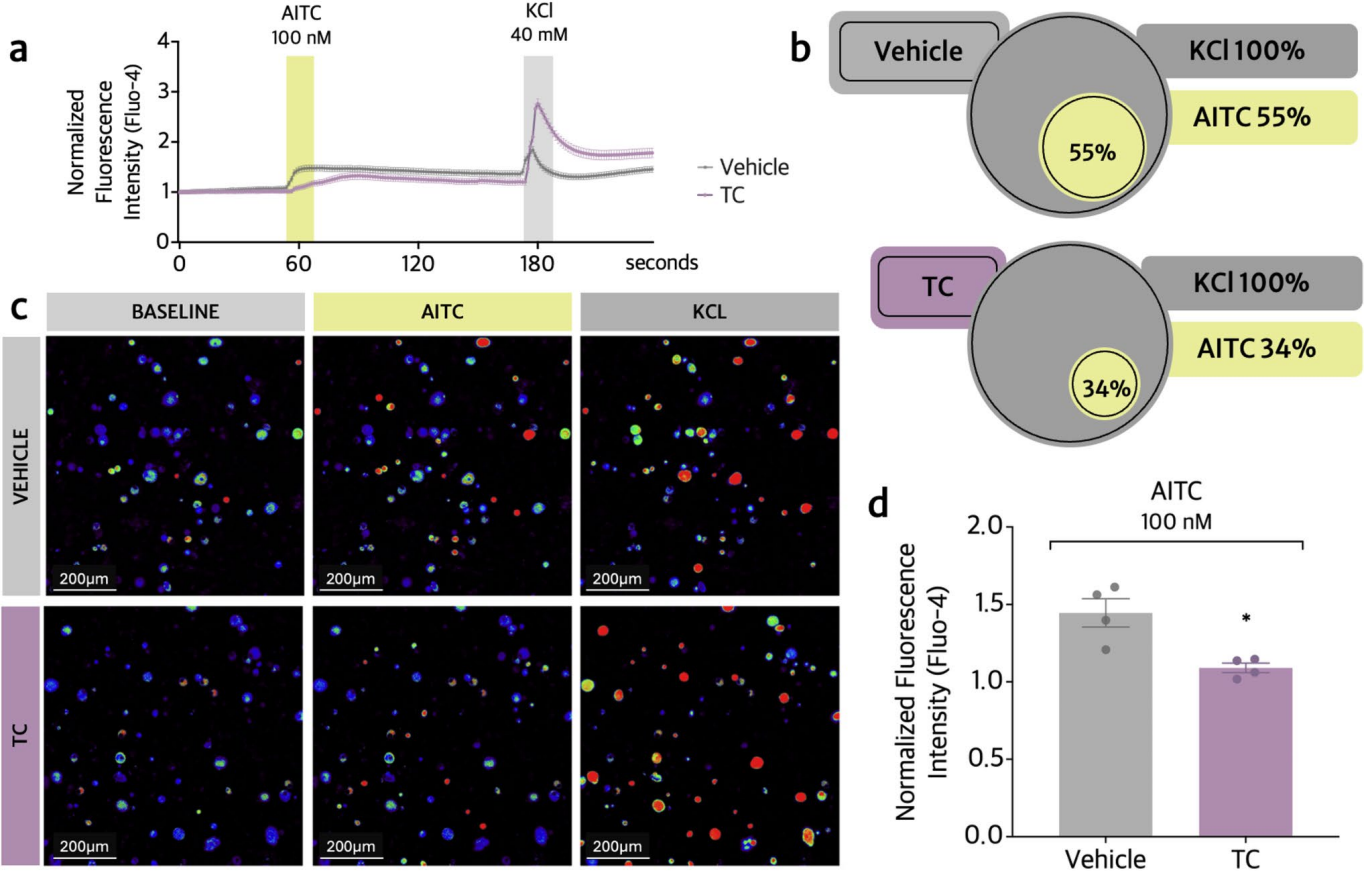
TC inhibits TRPA1^+^ nociceptive neuron activation in vitro. To assess TRPA1 activation in vitro, plates containing naïve DRG neurons were pretreated with TC (3 μM) or vehicle (HBSS 0.01% DMSO) for 60 min. Fluo-4-labeled cells were recorded for 4 min: 1 min of initial reading, followed by stimulus with AITC (100 nM in HBSS) for 2 min at the 60 s-mark, and with KCl for 1 min at the 180 s-mark (40 mM, activates all neurons). **a** Normalized fluorescence intensity tracers throughout the 4 min of recording. **b** Venn’s diagram indicating the percentage of DRG-derived neurons with increased calcium influx after AITC stimulus. **c** Representative images acquired at baseline, AITC stimulus, and KCl activation, for TC and vehicle groups. Captured with 20× objective lens. Scale bars = 200 μm (**d**) Comparative mean fluorescence intensity between vehicle and TC groups after AITC stimulus. Results are provided as means ± SEM of four culture dishes per group and each culture dish was a pool of DRG from 5 mice (n = 4 culture dishes). Vehicle group = total of 252 neurons; TC group = total of 160 neurons. Statistical analyses were performed by Shapiro–Wilk, and unpaired t test (Table 1). *p ≤ 0.05 compared to vehicle group

We also investigated whether TC could inhibit the activation of TRPV1^+^ (capsaicin-responsive) and TRPA1^+^ (AITC-responsive) DRG neurons. For this purpose, mouse DRG-derived primary neurons were stimulated with capsaicin (Fig. 7) or AITC (Fig. 8).

Pretreatment with TC (3 μM) effectively inhibited capsaicin-induced calcium influx compared to vehicle-treated DRG neurons, which can be observed through the reduced fluorescence intensity (20%) after adding the TRPV1 agonist (Fig. 7a–d) and the number of neurons activated by capsaicin, presenting a 36% rate of calcium influx after TC treatment, which was 30% lower than the control group, presenting a 51% calcium influx rate after vehicle treatment (Fig. 7b).

Neurons pretreated with TC (3 μM) and activated by the TRPA1 agonist AITC showed a decrease in fluorescence intensity (24%) (Figs. 8a–d) as well as a reduction in the percentage of neurons that underwent calcium influx upon the addition of AITC. There was an activation rate of 34% of viable neurons after TC treatment compared to a 54.5% activation rate for vehicle-treated DRG neurons, which represents an overall reduction of 37.6% in calcium influx due to TC treatment (Fig. 8b).

### TC alleviates CFA-induced hyperalgesia and inflammation, promotes hepatoprotection and shows no signs of stomach lesion

The experimental design using overt pain-like behavior with established overt pain-like behavior models was intended to focus on acute responses that depend on TRP channel activation (Figs. 3, 4, 5). These experiments were summed up with the aid of capsaicin and AITC as nociceptive stimuli because they are agonists of and activate TRPV1 and TRPA1, respectively (Figs. 6, 7, 8) (Caterina et al. 1997 and 2000; Bandell et al. 2004; Bautista et al. 2006). Nevertheless, we also expanded this investigation by assessing the activity of TC in a model with prolonged inflammation and other inflammatory and nociceptive responses as well as toxicity assays. We used the CFA paw inflammation model (Fig. S2). TC treatment started 24 h after CFA (10 μL) i.pl. administration (Fig. S2a). TC (10 mg/kg, p.o.) reduced CFA-triggered mechanical hyperalgesia (Fig. S2b) and thermal hyperalgesia (Fig. S2c). In addition to reducing pain, TC reduced paw edema (Fig. S2d), paw skin MPO (Fig. S2e) and NAG (Fig. S2f) activities, indicating an inhibitory effect over inflammatory parameters and recruitment of neutrophils and macrophages (Pinho-Ribeiro et al. 2015). Furthermore, TC reduced the plasma levels of AST (Fig. S2g) and ALT (Fig. S2h) indicating a reduction of CFA-triggered hepatic lesion/ inflammation, which is consistent with other findings that TC inhibits liver damage (Javadi et al. 2023; Ale-Ebrahim et al. 2022; Munakarmi et al. 2024; Singh et al. 2016; Jabbar et al. 2024; Karimi-Sales et al. 2018a). TC did not alter stomach MPO activity, a parameter that if increased would indicate tissue damage akin to the ulceration caused by non-steroidal anti-inflammatory drugs (Takeuchi 2012). These results present a perspective of TC therapeutic application in the treatment of prolonged inflammation as for the CFA model. In this scenario, repetitive TC treatment did not induce hepatic or stomach lesions. In fact, TC reduced the liver inflammation caused by CFA.

## Discussion

The primary goal of our study was to determine whether TC could be effective as an analgesic and whether its pharmacological activity would depend, at least in part, on inhibiting the activation of ion channels involved in overt pain-like behavior. The present data demonstrate that TC reduces overt pain-like behavior triggered by varied stimuli whose nociceptive mechanisms depend on activating, at least, TRPV1 and/or TRPA1 to cause peripheral nociceptive neurons activation. Explaining such action, TC reduced the nociceptive behavior and the activation of dorsal root ganglia neurons caused by agonists of TRPV1 and TRPA1. Therefore, although this seems not to be the exclusive analgesic mechanism of TC, the present results are the first to demonstrate a neuronal mechanism for TC by inhibition of TRPV1 and TRPA1 activation. The results were expanded in the CFA prolonged inflammation model, which demonstrated that TC treatment during a week reduced hyperalgesia and inflammation without causing liver and stomach lesion.

The writhing response to acetic acid and PBQ was the starting point to assess the flavonoid potential regulating nociception. The overt pain-like behavior induction by acetic acid and/or PBQ are widely used and effective to screen potential analgesics (Le Bars et al. 2001). Multiple mechanisms seem to underlie their nociceptive actions such as the production of prostanoids and cytokines (Ribeiro et al. 2000; Cunha et al. 2000; Verri et al. 2008). It is also noteworthy the involvement of TRP channels. The acetic acid-triggered writhing response is dependent on TRPV1 since TRPV1 deficient mouse exhibited almost a full blockade of abdominal contortions without effect of TRPA1 deficiency and without additive effects of double TRPV1/TRPA1 deficiency (Liu et al. 2013), possibly due to the sensing of low pH by TRPV1 (Heber et al. 2020; Dhaka et al. 2009). The ionic channels involved in PBQ-induced overt pain-like behavior remain unknown.

The formalin test is one of the best-characterized models for overt pain-like behavior testing, and its dual phase physiopathology is used to screen compounds with analgesic and/or anti-inflammatory potential due to the time-dependent sensitivity for different classes of analgesic drugs (Hunskaar et al. 1986; Hunskaar and Hole 1987). As the test comprises two phases, the initial phase (0–5 min) is accepted as the result of peripheral nociceptive neuron activation, which can occur via histamine H1 receptors and 5-hydroxytryptamine receptors HT-4/3 (Parada et al. 2001). Our results indicate TC ability to inhibit this signaling process. In the second phase (10–30 min), which was also inhibited with TC pretreatment, there is also a role for histamine H1 receptors and 5-hydroxytryptamine receptors HT-1A (Parada et al. 2001). Moreover, there are other inflammation-driven mechanisms, relying on cytokine signaling as well as the activation of ionic channels. IL-33, TNF-α, IL-1β, IL-8, and IL-6 cytokines have been shown to participate on the second phase of the formalin test (Magro et al. 2013; Chichorro et al. 2004). Thus, both phases of formalin test are dependent on the peripheral activation of primary afferent nociceptive neurons. The reduction observed in the second phase after TC treatment is coherent with previous findings in MSU-induced arthritis demonstrating TC inhibits cytokine production (Staurengo-Ferrari et al. 2018), which is the only evidence of TC effects in mechanical hyperalgesia inhibition described thus far. There are also central nervous system mechanisms activated by peripheral formalin injection with the participation of neurons, glial cells, cytokines and ion channels (McIlwrath et al. 2024; Wang et al. 2023a, b; Pavao-de-Souza et al. 2012). TC activity in the central nervous system remains to be addressed.

Another breakthrough mechanism described for the formalin test is the activation of TRPA1 channels in DRG neurons (McNamara et al. 2007). Data demonstrated that formalin induces the increase of calcium levels in HEK-293 cells expressing the human or the rat TRPA1. Formalin increase in neuronal calcium levels paralleled the response triggered by AITC and was inhibited by HC030031, a TRPA1 antagonist. Formalin IC50 is 1.2 μM and of AITC is 0.7 μM. Formalin activation of neurons was absent in TRPA1 deficient cells and HC030031 inhibited formalin nociceptive response (McNamara et al. 2007). Considering that the nociceptive mechanism of formalin depends on TRPA1 activation and TC inhibited formalin nociception, TC analgesic mechanism could involve TRPA1 inhibition.

Regarding CFA-induced nociception and hyperalgesia, the involvement of both TRPV1 and TRPA1 have been previously described. When TRPV1 was described to be the receptor activated by capsaicin, data demonstrated that CFA heat hyperalgesia was reduced in TRPV1 deficiency mouse (Caterina et al. 2000). Others have demonstrated applying pharmacological approaches that TRPV1 and TRPA1 participate in the nociceptive responses in the CFA model. For instance, TRPV1 antagonists (BCTC and SB-366791) administrated by intrathecal and i.pl. routes reduce CFA heat hyperalgesia (Kanai et al. 2007). The TRPA1 inhibitor called AP18 reduces neuronal activation, and the time spent licking the paw caused by the TRPA1 agonist cinnamaldehyde as well as the mechanical hyperalgesia triggered by CFA and bradykinin (Petrus et al. 2007). Summing up with the TC inhibition of the nociceptive responses triggered by acetic acid, PBQ and formalin, TC inhibited CFA-induced overt pain-like behavior. Recent data demonstrated that TC reduces CFA triggered production of cytokines, which can also account to explain the TC activity (Jabbar et al. 2024). Other mechanisms such as the role of TRP channels or other families of ion channels were not assessed by Jabbar et al. (2024).

Considering the results discussed so far, we reason that there was substantial evidence that TC can inhibit nociceptive behaviors that depend, at least in part, on TRPV1 and TRPA1 activation in nociceptive sensory neurons. Therefore, to further examine this potential mechanism, paw flinching and paw licking were induced in vivo by intraplantar injection capsaicin, an agonist of TRPV1; or AITC, an agonist of TRPA1. Summing up with the behavior assessment, primary cultures of axotomized DRG neurons from naïve mice were used to observe that capsaicin and AITC induce calcium flux in DRG neurons and TC can inhibit such responses. The in vitro experimental approach is important to narrow TC effects upon neuronal cells, demonstrating that its analgesic effects were, at least in part, resultant of the inhibition in TRPV1^+^ and TRPA1^+^ nociceptors activity. In agreement with our results, in silico evidence supports that other chalcones can bind to TRPV1 although it has not been verified if these compounds could actually function as TRPV1 antagonists (Benso et al. 2019). In vitro evidence has demonstrated that eleven chalcone derivatives, TC not included, induce calcium influx in HEK-293 cells expressing TRPA1 (Moriello et al. 2016). The present results present an additional approach since we used primary DRG neurons that already express TRPV1 and TRPA1. Some of those eleven chalcones were also tested in vivo using local treatment (topical eye administration) and induced eye wiping response. At least one of the chalcone derivatives caused more eye wiping than mustard oil (a TRPA1 agonist). The i.pl. treatment with these chalcones inhibited paw nociceptive behaviors caused by formalin. These results suggest that some chalcone derivatives can activate TRPA1 causing its desensitization and consequently reduce the nociceptive behavior caused by other algogenic stimuli (Moriello et al. 2016). Importantly, we observed in vitro that TC did not induce calcium influx in DRG neurons at a concentration of 3 μM that can inhibit capsaicin and AITC neuronal activation. These results suggest that TC mechanisms might not involve TRP channel desensitization, but rather antagonism, which is preferable since analgesia would not depend on initial activation as occurs with capsaicin topical application (Fattori et al. 2016).

It is consensus in the literature that the TRP channels V1 and A1 are involved in nociceptive responses (McNamara et al. 2007; Maximiano et al. 2024). This is because around 54% of DRG neurons are TRPV1^+^ and around 22% of DRG neurons are TRPA1^+^ while they sense varied noxious stimulation that cause nociceptive behavior (Bautista et al. 2005). Quite interestingly, DRG neurons can express both TRPV1 and TRPA1 and they also dimerize during nociceptive neuron sensitization (Bautista et al. 2005). The remaining subpopulation of TRPA1^+^/TRPV1^−^ DRG neurons are not involved in inflammatory pain, but rather in neuropathic pain (Patil et al. 2019). Under physiological conditions, once specific noxious stimulus to which TRPV1 and/or TRPA1 ionic channels are responsive come in contact with the sensory nerve fibers, they activate these receptors and allow ion influx to transduce the noxious stimulus into electrical activity, which is conducted to the DRG and spinal cord, where the signal is further propagated by synapse from the spinal cord to the brain (Jordt et al. 2003; Bautista et al. 2006; Dubin and Patapoutian 2010). Under pathological conditions, the continuous activation of neural nociceptive circuits induce functional plasticity such as transcriptional and post-translational modifications, leading to changes in both neuronal excitability and synaptic transmission, structural remodeling and reorganization of neural networks (Fiore et al. 2023). Therefore, the possibility of inhibiting both TRPV1 and TRPA1 ionic channels with a single compound is encouraging for acute pain as well as sustained painful conditions. In this sense, the present results were also expanded by applying the CFA paw inflammation model. TC post-treatment inhibited the hyperalgesia (mechanical and heat) caused by CFA in a protocol of 7 days. TC also inhibited inflammatory parameters such as paw edema, and the recruitment of neutrophils and macrophages as assessed by MPO and NAG activities. CFA injection in the paw also induces liver lesion (Oscherwitz et al. 2005), which was observed as an increase of plasma AST and ALT levels, inhibitable by TC. MPO activity increases in the stomach upon non-steroidal anti-inflammatory drugs treatment (Wallace et al. 1995), however, TC did not cause this response in the stomach. Therefore, these data demonstrate that TC reduces pain and inflammation and is safe in a 7-days treatment protocol because it does not cause liver and stomach lesions but rather treat CFA-triggered liver lesion.

It is worth discussing further that TC analgesic mechanisms might not be exclusively dependent on TRP channels inhibition, but rather TC might be a multi-target compound. Previous studies demonstrated its anti-inflammatory properties by inhibiting the production of cytokines such as IL-1β, TNF-α, IL-6, IL-12, IL-17, IL-18, and IFN-γ (Martinez et al. 2017a and 2017b; Staurengo-Ferrari et al. 2018; Karimi-Sales et al. 2018a; Jabbar et al. 2024; Cheng et al. 2024; Wang et al. 2024; Munakarmi et al. 2024). TC upregulates IL-10 and TGF-β production, which have anti-inflammatory and analgesic effects (Staurengo-Ferrari et al. 2018; Jabbar et al. 2024; Cheng et al. 2024). Moreover, TC inhibited other inflammatory markers such as the expression of the enzyme COX-2, the activation of the transcription factor NF-κB and STAT3 (Signal transducer and activator of transcription 3), NLRP3 inflammasome function, myeloperoxidase activity, the expression of adhesion molecules such as ICAM (intercellular adhesion molecule) and VCAM (vascular cell adhesion molecule), VEGF (vascular endothelial growth factor) production, and MMP9 (matrix metalloproteinase 9) activity (Lamoke et al. 2011; Martinez et al. 2017a and 2017b; Staurengo-Ferrari et al. 2018; Karimi-Sales et al. 2018a; Senrung et al. 2023; Jabbar et al. 2024; Cheng et al. 2024; Wang et al. 2024; Munakarmi et al. 2024). These mechanisms explain how TC can inhibit immune cell activation and migration, as well as contribute to tissue repair.

There is evidence that TC inhibits oxidative stress. TC is able to reduce ROS levels by inhibiting lipid peroxidation and superoxide anion production, possibly due to gp91^phox^, iNOS (inducible Nitric Oxide Synthase), and Keap1 (Kelch-like ECH-associated protein 1) downregulation, while TC antioxidant properties were observed on ferric reducing ability potential (FRAP) and ABTS (2,2′-azino-bis(3-ethylbenzothiazoline-6-sulfonic acid) assays, by catalase and glutathione increase, possibly related to Nrf2, heme oxygenase-1, and sirtuin 1 upregulation (Martinez et al. 2017a and 2017b; Staurengo-Ferrari et al. 2018; Karimi-Sales et al. 2018b; Miranda-Sapla et al. 2019; Komoto et al. 2021; Ale-Ebrahim et al. 2022; Cheng et al. 2024; Jabbar et al. 2024; Munakarmi et al. 2024; Wang et al. 2024). TRPV1 and TRPA1 can sense reactive oxygen species (Ma et al. 2009; Stanford et al. 2019). This evidence may initially suggest that TC inhibition of TRPV1 and TRPA1 activation could involve targeting oxidative stress. However, TC does not present chemical groups conferring direct antioxidant activity as well as it does not present antioxidant activity in cell free systems (Martinez et al. 2017a). Therefore, although TC belongs to the chalcone family of flavonoids, it is an atypical flavonoid because it is not antioxidant per se. This characteristic also makes TC an invaluable pharmacological tool to investigate the biological activities of flavonoids that are independent of intrinsic antioxidant chemical groups. Therefore, it is unlikely that TC inhibition of TRPV1 and TRPA1 activation depends on direct chemical structure antioxidant mechanisms. This conclusion supports that inhibition of TRPV1 and TRPA1 by flavonoids may also occur independently of intrinsic antioxidant chemical groups.

It is also important to mention the limitations of the present study. We selected a treatment regimen that has been used before (Staurengo-Ferrari et al. 2018) while additional data on TC pharmacokinetics would be interesting to further understand its activity. Although the direct treatment with TC in culture DRG neurons indicate that this compound is per se active, it is possible that as for other flavonoids (Nakamura et al. 2016; Sanechika et al. 2021; Rossato et al. 2010), in vivo metabolization may produce additional active compounds and we have not assessed that. Our study did not verify if TC can directly bind to TRPV1 and TRPA1. Our study was focused on overt pain-like behaviors of paw flinching, paw licking, and abdominal contortions because these behaviors depend on neuronal activation. TC also reduced mechanical and heat thermal hyperalgesia as a complementary data that align with prior published results (Jabbar et al. 2024). Hyperalgesia depends on nociceptive neuron sensitization involving other steps of neuronal and glia plasticity that also require TRP channels (Pinho-Ribeiro et al. 2016), which we did not approach herein. Evidence supports that TRPA1 is necessary for mechanical and cold hyperalgesia (Kwan et al. 2006). Furthermore, TRPA1 and TRPV1 interaction is also involved in cold hyperalgesia (Wang et al. 2023a, b). TRPA1 and TRPV1 form a complex in which TRPV1 activation increases TRPA1 activity when there is a third component in the complex, the transmembrane protein 100 (Tmem100) while in the absence of Tmem100, TRPV1 reduces TRPA1 activation (Fattori et al. 2016). Whether TC can affect TRPV1-TRPA1-Tmem100 complex and cold hyperalgesia remains to be investigated. The formalin and CFA models present central nervous system pathophysiological mechanisms, indicating that potential central mechanisms of TC can be further explored.

## Conclusions

The flavonoid TC has demonstrated multitargeted effects on multiple cell types (Staurengo-Ferrari et al. 2018; Sikander et al. 2011; Silva et al. 2018; Komoto et al. 2021). We have shown here, for the first time, that TC has analgesic properties that depend, at least in part, on inhibiting the activation of TRPV1^+^ and TRPA1^+^ nociceptive neurons. These data were obtained by the combination of in vivo and in vitro approaches, including a wide array of classical overt pain-like behavior models, TRPV1 and TRPA1 selective agonists and calcium imaging of cultured mouse primary DRG neurons. The present data adds to the existing literature on the analgesic role of TC by demonstrating a hitherto unrecognized neuronal mechanism of action that might influence on its future application and development as an analgesic.

## Supplementary Information

Below is the link to the electronic supplementary material.

Supplementary file1 (DOCX 35684 kb)

## Data Availability

Data is provided within the manuscript.

## Abbreviations

ABTS: 2,2′-Azino-bis(3-ethylbenzothiazoline-6-sulfonic acid)
AITC: Allyl isothiocyanate
ALT: Alanine transaminase
ANOVA: Analysis of variance
ARE: Antioxidant responsive elements
ARRIVE: Animal research: reporting of in vivo experiments
AST: Alanine transaminase
CFA: Complete Freund’s adjuvant
DMEM: Dulbecco’s modified eagle medium
DMSO: Dimethyl sulfoxide
DRG: Dorsal root ganglia
FRAP: Ferric reducing ability potential
GDNF: Glial cell line-derived neurotrophic factor
HBSS: Hanks’ balanced salt solution
ICAM: Intercellular adhesion molecule
iNOS: Inducible nitric oxide synthase
i.p: Intraperitoneal
Keap1: Kelch-like ECH-associated protein 1
MMP9: Matrix metalloproteinase 9
MPO: Myeloperoxidase
NAG: N-acetylglucosaminidase
NBM: Neurobasal medium
NADPH: Nicotinamide adenine dinucleotide phosphate
NF-κB: Nuclear factor κB
NLRP3: NOD-like receptor protein 3
NGF: Nerve growth factor
Nrf2: Nuclear erythroid 2-related factor
PBQ: Phenyl-*p*-benzoquinone
p.o.: Per oral
ROS: Reactive oxygen species
SEM: Standard error mean
STAT3: Signal transducer and activator of transcription 3
TC: *Trans*-Chalcone
TRPA1: Transient receptor potential ankyrin 1
TRPV1: Transient receptor potential vanilloid 1
VCAM: Vascular cell adhesion molecule
VEGF: Vascular endothelial growth factor
