# Supplementary material for: Trans-Chalcone alleviates overt pain-like behavior by targeting the activation of nociceptive neuron TRPV1 and TRPA1 channels
Source: Inflammopharmacology. 2026 Jan 8;34(2):1213–32. doi: 10.1007/s10787-025-02099-w (PMC12923438; doi:10.1007/s10787-025-02099-w)

**Supplementary information**

***Trans*-Chalcone alleviates overt pain-like behavior by targeting the activation of nociceptive neuron TRPV1 and TRPA1 channels**

**Inflammopharmacology**

Maiara Piva^1^, Kelly M. Yaekashi^1^, Thais G. O. Pereira^1^, Mariana M. Bertozzi^2^, Felipe A. Pinho-Ribeiro^1,3^, Cássia Calixto-Campos^1^, Doumit Camilios-Neto^4^, Sergio M. Borghi^1,5^, Ana C. Zarpelon-Schutz^1,6^, Victor Fattori^1,7^, Rubia Casagrande^2^, Waldiceu A. Verri, Jr ^1^*

^1^Department of Immunology, Parasitology and General Pathology, Center of Biological Sciences, Londrina State University, Londrina, Paraná, Brazil.

^2^Departament of Pharmaceutical Sciences, Center of Health Sciences, Londrina State University, Londrina, Paraná, Brazil.

**^3^**Division of Dermatology, Department of Medicine, Washington University School of Medicine in St. Louis, Saint Louis, Missouri, United States.

^4^Department of Biochemistry and Biotechnology, Centre of Exact Sciences, Londrina State University, Londrina 86057-970, PR, Brazil.

^5^Center for Research in Health Sciences, University of Northern Paraná, Londrina, Paraná, Brazil.

^6^Campus Toledo, Universidade Federal do Paraná, Toledo 85919-899, Paraná, Brazil.

^7^Departament of Vascular Biology Program, Department of Surgery, Boston Children’s Hospital-Harvard Medical School, Karp Research Building, Boston, Massachusetts, United States.

*Correspondence: Prof. Waldiceu A. Verri Jr., Biological Sciences Center, Londrina State University. Rod. Celso Garcia Cid Pr 445 KM 380, Londrina, Paraná, Brazil. P.O. box 10.011, zip code 86057-970. Phone: +55 43 3371-4979, Faz: +55 43 3371-4387, [waldiceujr@yahoo.com.br](mailto:waldiceujr@yahoo.com.br) or [waverri@uel.br](mailto:waverri@uel.br).


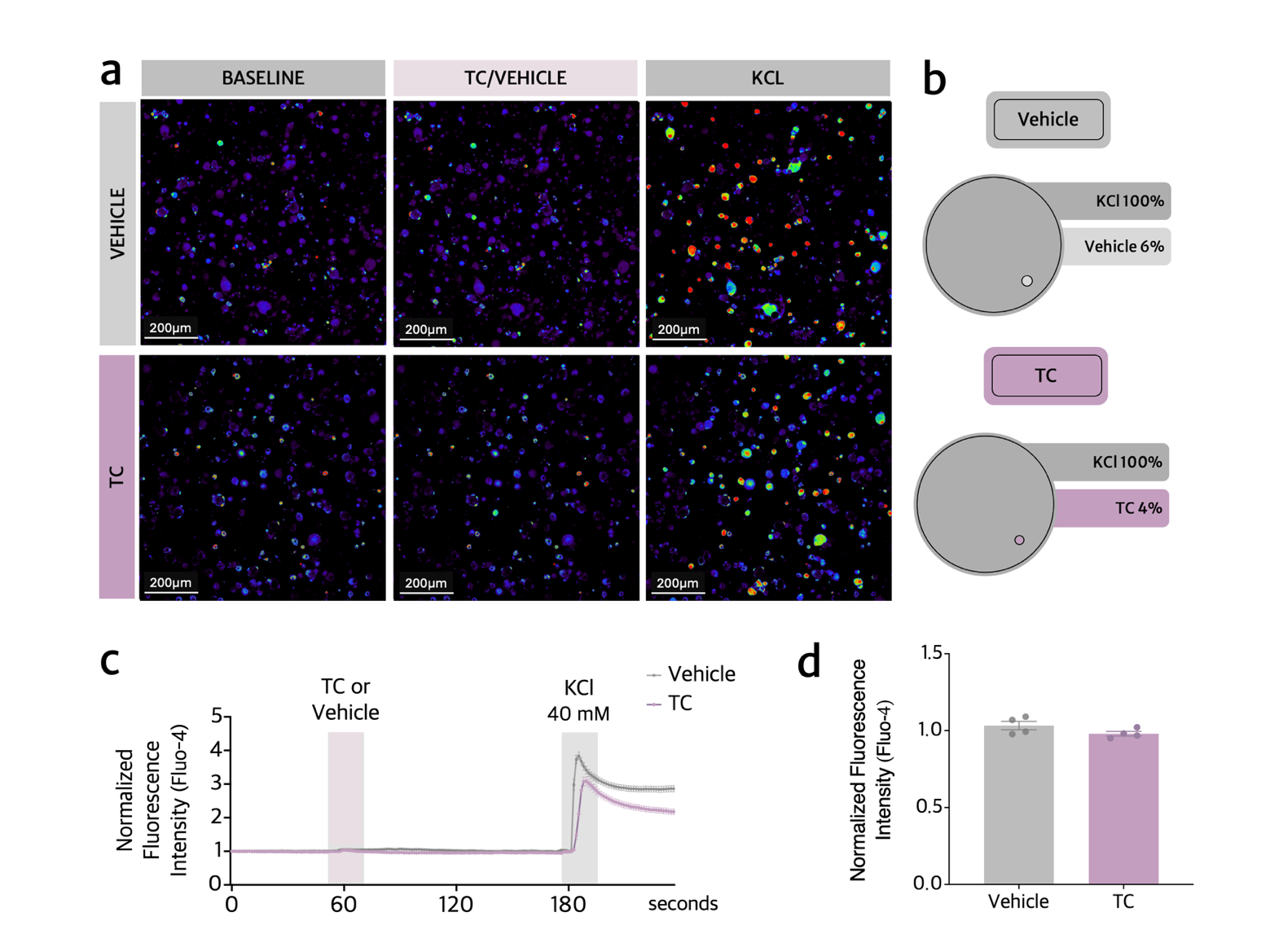


**Fig. S1** *TC does not promote neuronal calcium influx in vitro.* To assess potential TC-induced activation of primary DRG neurons in vitro, culture plates seeded with naïve neurons loaded with the fluorescent probe Fluo-4 were recorded for 4 min: 1 min of initial reading, followed by treatment with TC (3 μM; Staurengo-Ferrari et al., 2018) or vehicle (HBSS 0.01% DMSO) for 2 min at the 60 s-mark, and with KCl for 1 min at the 180s-mark (40 mM, activates all neurons). (**a**) Representative images acquired at baseline, TC or vehicle treatment, and KCl activation, for both groups. Captured with 20x objective lens. Scale bars = 200 μm. (**b**) Venn’s diagram indicating the percentage of DRG neurons response upon TC, vehicle or KCl addition. (**c**) Normalized fluorescence intensity tracers throughout the 4 min of recording. (**d**) Comparative mean fluorescence intensity between vehicle and TC groups at baseline and after treatments addition. Results are provided as means ± SEM of four culture dishes per group and each culture dish was a pool of DRG neurons from 5 mice (n=4 culture dishes). Statistical analyses were performed by Shapiro-Wilk, and unpaired t test (**Table S1**).


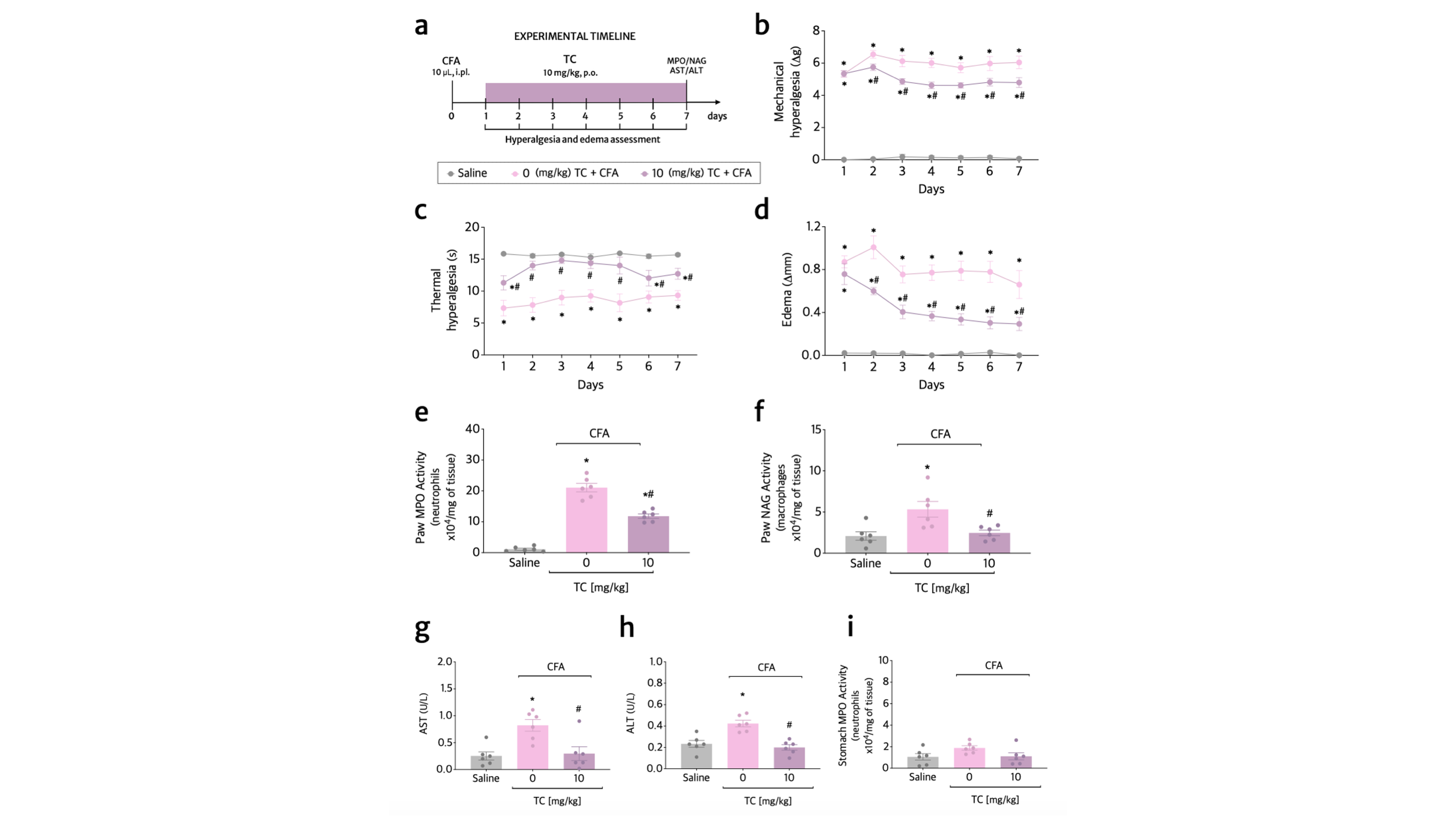


**Fig. S2.** *TC reduces CFA-induced hyperalgesia and inflammation, displays no sign of toxicity, and improves liver function.* (**a**) Experimental timeline. 24 h following the i.pl. stimulus with CFA (10μL), mice were treated orally with vehicle (20% Tween-80 Saline) or TC (10 mg/kg) 30 minutes before measurements. Daily treatments and behavior analyses were sustained until the 7^th^ day. Mechanical (**b**) and thermal (**c**) hyperalgesia were evaluated by electronic von Frey and hot plate, respectively. The inflammatory parameters edema formation (**d**) and leukocyte recruitment (MPO and NAG activities) to the CFA-induced plantar tissue (**e, f**) were investigated. Liver function markers AST (**g**) and ALT (**h**) were analyzed at the 7^th^ day post-CFA injection, as well as the stomach MPO activity (**i**). Results are presented as means ± SEM of six mice per group. Shapiro-Wilk and Brown-Forsythe tests were used to assess normality and equality of group variances, respectively. Statistical analyses were performed by one- or two-way ANOVA followed by Tukey’s multiple comparisons test (**Table S1**). *p ≤ 0.05 compared to saline group; ^#^p ≤ 0.05 compared to stimulus group.

**Table S1.** Summary of the statistical analyses of Figures S1 and S2.


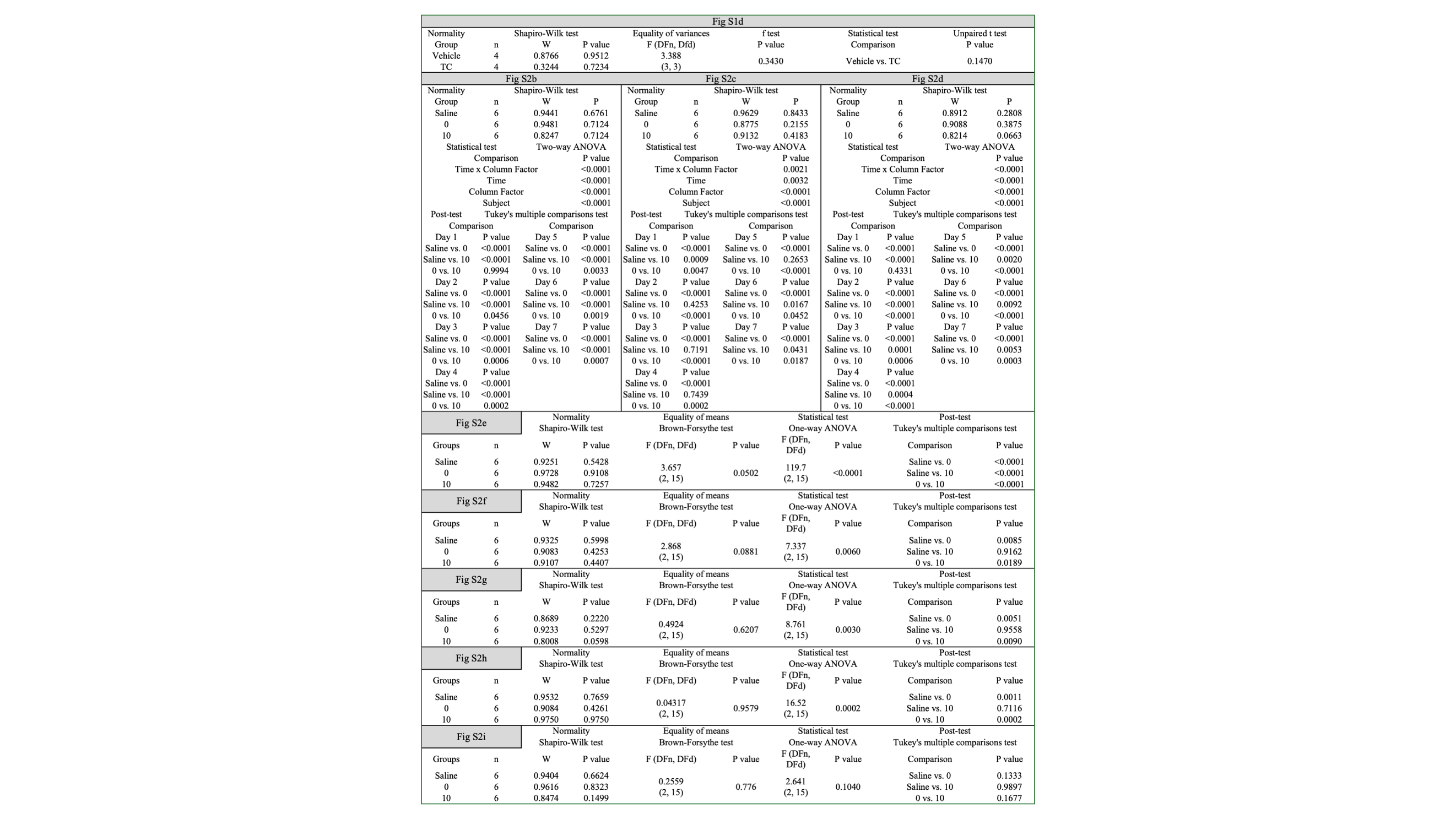

Supplement: Supplementary file 1 — Supplementary file1 (DOCX 35684 kb) [file 10787_2025_2099_MOESM1_ESM.docx]
